# Greater adherence to the Dietary Guidelines for Americans is associated with lower diet-related greenhouse gas emissions but higher costs

**DOI:** 10.3389/fnut.2023.1220016

**Published:** 2023-08-02

**Authors:** Zach Conrad, Adam Drewnowski, David C. Love

**Affiliations:** ^1^Department of Kinesiology, William & Mary, Williamsburg, VA, United States; ^2^Global Research Institute, William & Mary, Williamsburg, VA, United States; ^3^Center for Public Health Nutrition, University of Washington, Seattle, WA, United States; ^4^Department of Environmental Health and Engineering, Johns Hopkins Bloomberg School of Public Health, Johns Hopkins University, Baltimore, MD, United States; ^5^Center for a Livable Future, Johns Hopkins Bloomberg School of Public Health, Johns Hopkins University, Baltimore, MD, United States

**Keywords:** NHANES, Dietary Guidelines for Americans, Healthy Eating Index, popular diet, sustainability, greenhouse gas

## Abstract

**Introduction:**

Few studies have evaluated the sustainability of popular diet patterns in the US, which limits policy action and impedes consumer efficacy to make sustainable dietary changes. This study filled this gap by evaluating the relationship between diet quality, greenhouse gas emissions (GHGE), and diet cost for plant-based, restricted carbohydrate, low grain, low fat, and time restricted diet patterns.

**Methods:**

Dietary data were retrieved from the National Health and Nutrition Examination Survey (2011–2018, *n* = 8,146) and linked with data on GHGE and food prices from publicly available databases. Diet quality was measured using the Healthy Eating Index-2015. The present study (1) compared the mean diet quality, GHGE, and diet cost between diet patterns, (2) evaluated the association of diet quality to GHGE and diet cost for each diet pattern, and (3) estimated the contribution of food sources to GHGE and diet cost for each diet pattern.

**Results:**

Higher diet quality was associated with lower GHGE for the general population and for most diet patterns (*p* < 0.01) except for the plant-based and time restricted diet patterns (*p* > 0.05). Higher diet quality was associated with higher cost for the general population and for all dietary patterns (*p* < 0.01) except the time restricted diet pattern (*p* > 0.05). Protein foods, mostly beef, accounted for the largest share of GHGE (29–40%) and diet cost (28–47%) for all diet patterns except plant-based.

**Discussion:**

Higher diet quality was associated with lower GHGE but was often accompanied by higher diet cost. These sustainability trade-offs can help inform major policy discussions in the US and shed light on further research needs in the area of food systems transformation.

## 1. Introduction

Monetary and environmental costs of healthy diets are among the major challenges faced by the current food system (1). Most of the global population is unable to afford a healthy diet and there are growing concerns about equity and inclusion (2). Up to 11 million people die every year from suboptimal nutrition (3, 4), which is the leading modifiable risk factor for mortality (4). At the same time, food systems are responsible for up to 35% of greenhouse gas emissions (GHGE) and need to fit within planetary boundaries (5). Global policy agendas have been developed to address these challenges, including the United Nations Sustainable Development Goals for 2030, which includes targets for healthy diets, overall health and well-being, agricultural stewardship, management of natural resources, and safe working environments (6).

In the United States (US), dietary recommendations are met by only 5% of the population and nutrition disparities have worsened (7, 8). Suboptimal diets are now the leading cause of death (9), accounting for 45% of deaths from cardiometabolic diseases (10). The US food system accounts for 18% of annual GHGE but only 9% of the Gross Domestic Product (11). There are also concerns about whether healthy, affordable, and climate friendly diet patterns can be achieved by lower income minority groups (2, 12), and concerns about forced labor in the US food system have also risen (13). The National Strategy on Hunger, Nutrition, and Health, released in September 2022, proposes a whole-of-society approach to improve access to healthy, affordable, and climate friendly diet patterns (14). That initiative includes supporting research that explores relationships between nutrition, environment, affordability, and equity (Pillar 5) (14). Additionally, multiple US federal agencies have pledged to support new programs to bolster the evidence base for nutrition-sustainability relationships (15).

More than 50% of people in the US reported consuming a specific diet pattern at any point in 2022, up from 39% in 2021 (16). These include plant-based, low carbohydrate, intermittent fasting, and others. Nearly one-half of adults reporting following at least one specific diet pattern on a given day from 2005 to 2018, and almost 10% followed more than one diet pattern (17). Over one-third of adults reported that promoting health was their primary motivation for following one of these diet patterns and 21% reported that their primary motivator was improving environmental sustainability (16). Others have shown that dietary changes among people motivated by health and environmental concerns can reduce GHGE emissions by nearly 7% (18).

Few studies have evaluated the sustainability impacts of actual diet patterns from dietary surveys in the US (19–21), rather than theoretical (22–28) or self-categorized (18, 29) diet patterns. Recently, others have demonstrated that, among healthy vegetarian diet patterns, higher diet quality was associated with lower GHGE; but the same was not observed for unhealthy vegetarian diet patterns (19). In other studies, keto (≤50 g net carbohydrates) and paleo (<0.5 oz.-equivalents of grains and legumes and < 0.25 cup-equivalents of dairy) diet patterns were associated with higher GHGE and lower diet quality than some but not all plant-based diet patterns (20). Recently, others showed that plant-based diet patterns were associated with lower GHGE but their diet quality and cost were similar to other diet patterns (21).

No study has evaluated the relationships between diet quality, GHGE, and diet cost for restricted carbohydrate, low grain, low fat, and time restricted diet patterns. To address this research gap, the present study (1) compared the mean diet quality, GHGE, and diet cost between plant-based, restricted carbohydrate, low grain, low fat, and time restricted diet patterns, (2) evaluated the linear association of diet quality to GHGE and diet cost for each diet pattern, and (3) estimated the contribution of food sources to GHGE and diet cost for each diet pattern.

## 2. Methods

### 2.1. Dietary data

Data on dietary intake and sociodemographic characteristics at the individual level were acquired from the National Health and Nutrition Examination Survey (NHANES), 2011–2018 (30). NHANES uses a multi-stage sampling design to collect data continuously from approximately 5,000 non-institutionalized participants per year, and data are released in 2-years cycles (30). The computer-assisted Automated Multiple Pass Method is used to collect dietary data as part of the 24-h recall, and data from the first of two 24-h recalls were used because this appropriately measures *per capita* intake. The Food and Nutrient Database for Dietary Studies (FNDDS) (31) and Food Patterns Equivalents Database (FPED) (32) were used to categorize foods reported consumed by participants into food categories (Supplementary Table S1). The present study was exempted from human studies ethical review by the Institutional Review Board at William & Mary.

### 2.2. Diet pattern categorization

Data on daily food and nutrient intake were used to categorize participants into the following diet patterns: food group restricted (plant-based, low grain), macronutrient restricted (restricted carbohydrate, low fat), and time restricted. Participants that were categorized into more than one diet pattern were not included in the final analytic sample to establish mutually exclusive diet patterns, as described below. The operationalization of these diet patterns is described in Table 1 and Supplementary Table S2, and was informed by prior research (17, 33, 34). Data on food intake from NHANES was converted into food groups using the Food Patterns Equivalents Database (FPED) (32), and these data were used to categorize food group restricted diet patterns. NHANES also provides data on nutrient intake and the time between each eating occasion for each participant, and these were used to categorize macronutrient restricted and time restricted diet patterns, respectively.

**Table 1:** Criteria for diet pattern categorization.

Diet pattern	*n*	Inclusion criteria (daily intake)^a^
Food group restricted		
Plant-based	1,022	<1 ounce-equivalent of meat, poultry, and seafood
Low grain	740	≤25th percentile of total grain intake
Macronutrient restricted		
Restricted carbohydrate	3,529	<45% kcal from carbohydrate
Low fat	2,490	<30% kcal from fat
Time restricted	365	≥12 hours fast of food and beverages >0 kcal

a Data on daily nutrient intake were acquired from the nutrient intake files in the National Health and Nutrition Examination Survey, and data on daily intake of food groups were acquired from the Food Pattern Equivalents Database.

### 2.3. Diet quality measurement

Diet quality was measured using the Healthy Eating Index-2015 (HEI-2015) because it measures compliance with the Dietary Guidelines for Americans (35) and is therefore relevant to contemporary policy discussions in the US (14, 36). HEI-2015 includes nine components to encourage (total fruit, whole fruit, total vegetables, greens and beans, whole grains, dairy, total protein foods, seafood and plant proteins, and the ratio of unsaturated to saturated fats) and four components to limit (refined grains, sodium, added sugars, and saturated fats). Intake amounts for most components are energy-adjusted to 1,000 kcal and scored from 0 to 5 or 0 to 10 with higher scores being more favorable, and intermediate intakes are scored proportionally. Scores for all components are summed to estimate a total score for each participant out of a maximum score of 100 (35). The simple scoring algorithm with regression modeling was appropriately used to calculate HEI-2015 scores (37).

### 2.4. Diet cost

National average food prices for each NHANES food (2011–2018) were acquired from the Purchase-to-Plate Price Tool (PPPT), developed by the US Department of Agriculture’s Economic Research Service (USDA ERS) (38). Data were provided to USDA ERS under contract with Information Resources, Inc., which derives the data from InfoScan, which represents nearly 50% of all retail food sales in the US (39). InfoScan is a program that collects transaction data from retail checkout scanners across the US, and USDA ERS adjusts the food prices for losses and waste to reflect the cost associated with the consumed portion only, and uses machine learning to match these data with foods consumed by NHANES participants (40).

NHANES participants indicate whether they consumed each food at home (FAH) or away from home (food away from home, FAFH), yet data from PPPT only represent FAH prices and there are no publicly available data on national average FAFH prices for each food reported consumed in NHANES. Therefore, USDA ERS applies PPPT prices to all foods reported consumed by NHANES participants. This will underestimate total food expenditure because consumers typically face higher prices for FAFH than FAH, and other data show that FAFH accounts for approximately 50% of consumer food expenditures (41). Therefore, the present study derived FAFH prices using a methodology previously demonstrated (42, 43) and described below.

Data on FAH and FAFH prices from the National Household Food Acquisition and Purchase Survey (FoodAPS) (44) were used to derive a coefficient that converted FAH prices (from PPPT) to FAFH prices for each of the FAFH reported consumed by NHANES participants. FoodAPS used a multi-stage survey design to collect information from US households from April 2012 through January 2013 on the price of FAH and FAFH from receipts and scanned barcodes (44). Survey-weighted mean FAH and FAFH prices were estimated for each major food group (meat, poultry, seafood, eggs, dairy, fats and oils, fruits and vegetables, sweets, grains, non-alcoholic beverages, and other foods), and these were used to derive a coefficient that represents the ratio of FAFH-to-FAH prices for each food group. These coefficients were multiplied by the price of each FAFH in PPPT to estimate its FAFH price. For example, if the price of a given dairy food was $2.35 (from PPPT), and if the mean price of FAFH dairy was 2.06 times greater than the mean price of FAH dairy (from FoodAPS), the adjusted price of that given dairy food would be estimated as $4.85 ($2.50 × 2.06).

### 2.5. Greenhouse gas emissions

The database of Food Impacts on the Environment for Linking to Diets (dataFIELD) (45) provided data on greenhouse gas emissions (GHGE) for each food reported consumed by NHANES participants. To create dataFIELD, a systematic review of food environmental life cycle assessments (LCA) published between 2005 and 2016 was performed (*n* = 321 studies), which resulted in 1,645 combinations of food types and production scenarios (46). Nearly all entries accounted for impacts from agricultural production, 51% accounted for impacts from food processing, 19% accounted for impacts from retail and regional food hubs, and 6% accounted for consumer-level impacts (46). Impact data for each food were averaged across studies and matched to commodities in the Food Commodity Intake Database (FCID), which provides information on the amount of approximately 500 ingredients in each food reported consumed by NHANES participants (47). FCID was established by the US Environmental Protection Agency (US EPA) but has not updated since 2010 so others have developed methods for updating these data to align with more recent NHANES surveys (48), which were used in the present study.

### 2.6. Retail loss, consumer waste, and inedible portions

Consumer food demand embodies the environmental impacts associated with the consumed portion as well as the portions lost and wasted at the retail and consumer levels, yet dataFIELD only provides the impacts associated with the consumed portion (46). Similarly, consumer food prices include the cost of the edible portion of food as well as the portions that are inedible and those that will eventually be wasted, yet PPPT only provides the prices associated with the edible portion (49). To fill these data gaps, the present study used methods developed by others to estimate the environmental impacts of Total Food Demand (TFD), which represents the sum of impacts from retail loss, inedible portions, consumer food waste, and consumed food (50, 51); as well as the cost of purchased food, which represents the sum of costs associated with consumer food waste, inedible portions, and consumed food (42, 43).

FCID was used to disaggregate each food reported consumed by NHANES participants into its constituent ingredients. Then each FCID ingredient was matched to a food commodity in the USDA Loss-adjusted Food Availability data system (LAFA) (52), which provides data on the amount of retail loss, consumer waste, and inedible portions associated with each commodity. These gram amounts for each ingredient for each loss and waste category in each NHANES food were multiplied by the GHGE per gram of each FCID ingredient (from dataFIELD), and the values for each loss and waste category were summed across all ingredients within each NHANES food. To estimate purchase prices, the gram amounts of each loss and waste category of each NHANES food were multiplied by the price per gram of each food (from PPPT). For each NHANES food, the environmental impacts associated with retail loss, consumer waste, inedible portions, and consumed food were summed to estimate the impacts attributable to TFD; and the costs associated with consumer waste, inedible portions, and consumed food were summed to estimate the cost attributable to purchased food. Additional details of the NHANES-FCID-LAFA linkage procedure, including sources of uncertainty and embedded assumptions, are described in detail elsewhere (43, 51).

### 2.7. Statistical analyses

Mean diet quality (consumed), GHGE (TFD), and diet cost (purchased) were estimated for each diet pattern, and for each food category within each diet pattern, using linear regression models adjusted for kcal (continuous) and survey cycle (continuous) to account for potential confounding from total energy intake and data collection period. Multivariable linear regression models were also used to evaluate the relationship between diet quality and each dependent variable (log-transformed GHGE and diet cost) for each diet pattern. Comparisons of means and regression coefficients between diet patterns were evaluated with Wald tests and statistical significance was set at *p* < 0.005 with an integrated Bonferroni correction for multiple comparisons (*p* < 0.05/10 comparisons = 0.005). Statistical significance of regression coefficients was also assessed for each diet pattern independently by testing the difference between each coefficient and zero using Wald tests with statistical significance set at *p* < 0.05. Four series of sensitivity analyses were used to evaluate the robustness of the results by (1) removing the adjustment for losses and waste, (2) removing the adjustment for FAFH prices, (3) including an adjustment for self-reported demographic variables including age (continuous), sex (male and female), education (<high school, high school or equivalent, some college, and college graduate), income-to-poverty-ratio (<1.30, 1.31–1.99, 2.00–3.99, and ≥ 4.00), and race and Hispanic origin (non-Hispanic white, non-Hispanic black, Hispanic including Mexican-American, and other including multi-racial), and (4) including participants that were categorized into >1 diet pattern. All analyses accounted for the multistage probability sampling design of NHANES using standardized procedures and variables provided by the National Center for Health Statistics. Stata16.1 (StataCorp; College Station, TX) was used for data management and analysis.

## 3. Results

### 3.1. Final sample estimation

A total of 33,325 respondents provided data on dietary intake from 2011 to 2018 (Supplementary Figure S1). Respondents were excluded if they were < 20 y (*n* = 13,719), pregnant or breastfeeding (*n* = 359), did not consume one of the diet patterns of interest (*n* = 4,172), consumed >1 diet pattern of interest (6,551), and had ≥1 sustainability impact (diet quality, GHGE, or diet cost) that was >3SD from the mean (*n* = 278). The final analytic sample included 8,146 respondents. For completeness, outcomes were also reported for the general population (*n* = 18,969), which included respondents that did not consume one of the diet patterns of interest and those that consumed >1 diet pattern of interest. The results for the general population are reported as footnotes in the tables and figures.

### 3.2. Participant characteristics

The greatest proportion of respondents in the plant-based diet group (*n* = 1,022) were 51–70 y (38%), female (66%), college graduates (38%), had an income-to-poverty ratio ≥ 4.00 (37%), and were non-Hispanic white (70%; Table 2). The greatest proportion of respondents in the low grain diet group (*n* = 740) were 31–50 y (35%), female (57%), completed some college (37%), had an income-to-poverty ratio of 2.00–3.99 (32%), and were non-Hispanic white (63%). In the restricted carbohydrate group (*n* = 3,529), the greatest proportion of respondents were 51–70 y (38%), male (54%), graduated college (39%), had an income-to-poverty ratio of ≥4.00 (46%), and were non-Hispanic white (72%). In the low fat group (*n* = 2,490), the greatest proportion of respondents were 51–70 y (35%), college graduates (31%), had an income-to-poverty ratio of ≥4.00 (35%), were non-Hispanic white (60%), and approximately half were female (51%). In the time restricted diet group (*n* = 365), the greatest proportion of respondents were 20–30 y (35%), male (58%), completed some college (42%), had an income-to-poverty ratio ≤ 1.30 (32%), and non-Hispanic white (48%).

**Table 2:** Characteristics of study participants, 2011–2018 (*n* = 8,146).

Characteristic	Food group restricted			Macronutrient restricted				Time restricted^e^ (*n* = 365)		
	Plant-based^a^ (*n* = 1,022)		Low grain^b^ (*n* = 740)	Restricted carbohydrate^c^ (*n* = 3,529)			Low fat^d^ (*n* = 2,490)			
	% (95% CI)									
Age, y										
20–30	17.0	(13.5, 21.2)	25.7	(20.9, 31.1)	15.9	(14.2, 17.8)	17.2	(14.3, 20.6)	35.1	(28.3, 42.5)
31–50	30.1	(25.5, 35.1)	34.6	(29.3, 40.3)	33.5	(30.5, 36.7)	34.3	(31.7, 37.0)	27.1	(20.5, 34.9)
51–70	37.6	(32.8, 42.7)	27.8	(22.9, 33.3)	38.1	(35.6, 40.6)	35.3	(31.9, 38.8)	24.9	(17.7, 33.7)
>70	15.3	(12.1, 19.1)	11.9	(8.9, 15.8)	12.5	(11.1, 14.0)	13.2	(11.4, 15.2)	13.0	(8.7, 18.9)
Sex										
Male	34.5	(30.0, 39.3)	43.0	(38.2, 47.9)	54.1	(51.5, 56.6)	48.8	(45.7, 51.8)	57.8	(49.3, 65.9)
Female	65.5	(60.7, 70.0)	57.0	(52.1, 61.8)	45.9	(43.4, 48.5)	51.2	(48.2, 54.3)	42.2	(34.1, 50.7)
Education										
<High school	12.6	(10.0, 15.9)	17.3	(14.1, 20.9)	9.3	(7.9, 10.9)	16.2	(14.1, 18.6)	16.5	(12.1, 22.2)
High school or equivalent	19.9	(16.3, 24.1)	27.1	(22.3, 32.5)	21.6	(19.5, 23.8)	22.9	(20.3, 25.7)	31.7	(24.9, 39.3)
Some college	29.1	(25.3, 33.3)	36.7	(32.9, 40.8)	30.6	(28.0, 33.4)	30.1	(27.5, 32.8)	41.5	(34.0, 49.4)
College graduate	38.3	(33.3, 43.6)	18.9	(15.2, 23.4)	38.5	(35.0, 42.2)	30.8	(27.6, 34.2)	10.3	(6.9, 15.1)
Income-to-poverty ratio										
≤1.30	24.4	(20.7, 28.5)	29.6	(25.2, 34.4)	15.9	(14.1, 17.8)	23.9	(20.6, 27.6)	32.3	(25.7, 39.8)
1.31–1.99	16.0	(13.3, 19.3)	14.2	(10.8, 18.6)	10.8	(9.4, 12.4)	14.5	(12.0, 17.4)	18.3	(13.2, 24.8)
2.00–3.99	22.6	(18.8, 27.0)	32.4	(27.3, 38.1)	27.4	(24.9, 30.1)	27.0	(24.0, 30.2)	30.4	(22.9, 39.1)
≥4.00	36.9	(31.5, 42.6)	23.8	(18.1, 30.5)	45.9	(42.4, 49.5)	34.6	(30.4, 39.1)	18.9	(12.8, 27.0)
Race and Hispanic origin^f^										
Non-Hispanic white	69.8	(64.5, 74.6)	63.0	(56.4, 69.1)	71.7	(68.4, 74.8)	60.4	(55.8, 64.8)	47.6	(38.8, 56.5)
Non-Hispanic black	5.0	(3.7, 6.7)	14.7	(11.6, 18.3)	9.4	(7.7, 11.4)	10.4	(8.6, 12.5)	24.0	(18.5, 30.6)
Hispanic^g^	14.0	(10.8, 17.8)	13.7	(10.4, 17.8)	11.9	(9.8, 14.3)	18.7	(15.8, 21.9)	20.9	(14.7, 28.8)
Other^h^	11.2	(8.2, 15.1)	8.7	(6.6, 11.3)	6.9	(5.8, 8.4)	10.6	(8.5, 13.1)	7.5	(4.6, 12.1)

Sample sizes are unweighted.

a <1 ounce-equivalent of meat, poultry, and seafood.

b ≤25th percentile of total grain intake.

c <45% kcal from carbohydrate.

d <30% kcal from fat.

e ≥12 hours food and beverage fast.

f Self-identified based on the interview prompts “Do you consider yourself to be Hispanic, Latino, or of Spanish origin?”, “Please give me the number of the group that represents your Hispanic/Latino or Spanish origin or ancestry”, and “What race do you consider yourself to be [check all that apply]?”

g Includes Mexican American.

h Includes multi racial.

### 3.3. Mean daily diet quality, greenhouse gas emissions, and diet cost

**Figure 1:**
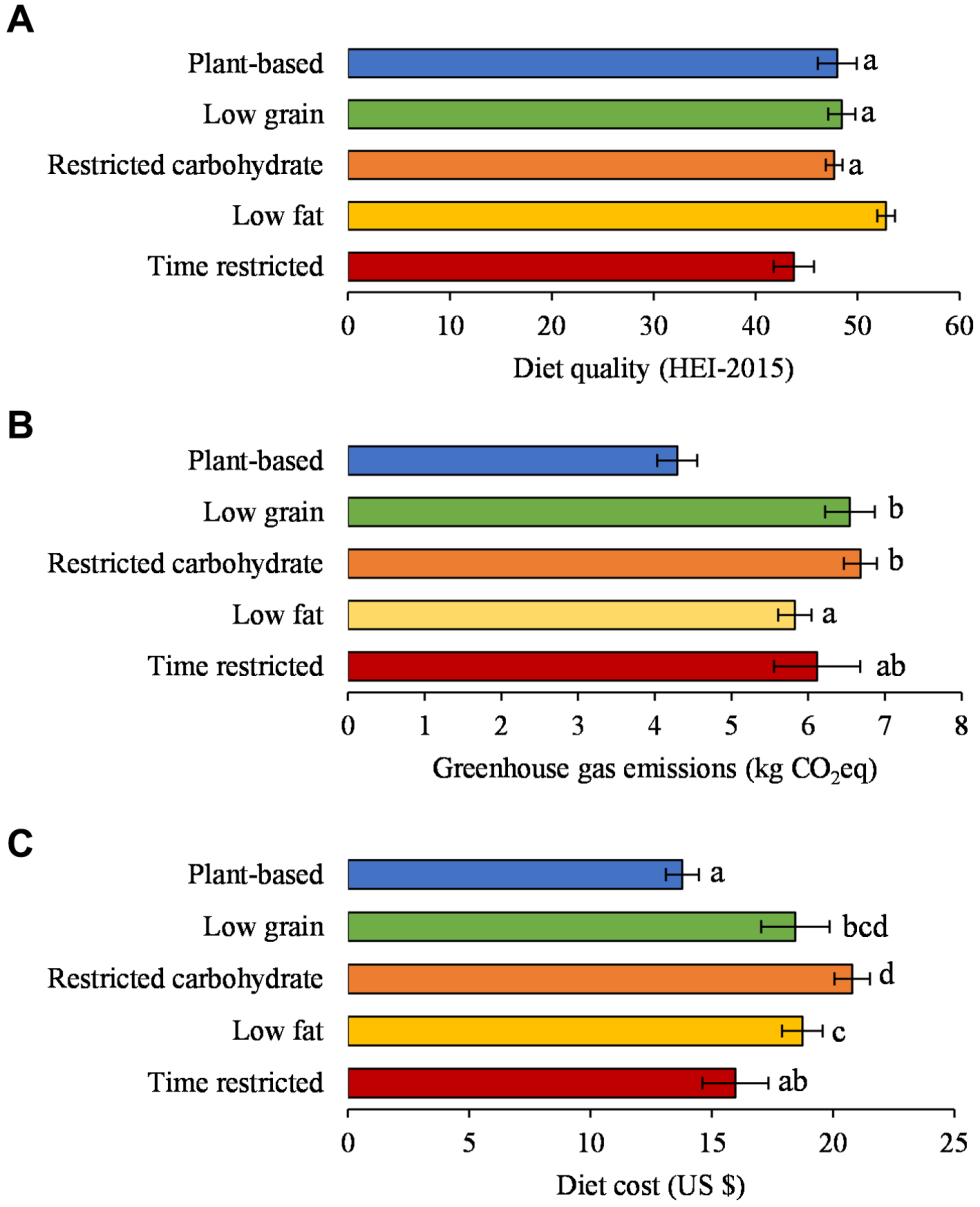
Mean daily **(A)** diet quality, **(B)** greenhouse gas emissions, and **(C)** diet cost of popular diet patterns, 2011–2018 (*n* = 8,146). Diet patterns sharing a letter are not statistically different at *p* < 0.005 using Wald tests (Bonferroni correction: 0.05 ÷ 10 pairwise tests = 0.005). All results were adjusted for kcal and survey cycle using linear regression models. HEI-2015, Healthy Eating Index-2015. CO_2_eq, carbon dioxide equivalent. General population (all adults categorized into a popular diet pattern and those not categorized and met all inclusion criteria, *n* = 18,969): diet quality: 49.0 (95%CI: 48.4–49.5); greenhouse gas emissions: 6.1 kg CO_2_eq (95%CI: 6.0–6.2 kg CO_2_eq); diet cost: $18.87 (95%CI: $18.42–$19.32).

The low fat diet pattern had the highest diet quality score (52.8, 95% CI: 52.0, 53.7) and the time restricted diet pattern had the lowest diet quality score (43.7, 95% CI: 41.8, 45.7; Figure 1A). The diet quality scores for the plant-based (48.0, 46.1, 49.9), low grain (48.4, 47.1, 49.8), and restricted carbohydrate (47.7, 46.9, 48.5) diet patterns were similar. The diet quality score for the general population was 49.0 (48.4, 49.5). The plant-based diet pattern had the lowest GHGE (4.3 kg CO_2_eq, 95% CI: 4.0, 4.6 kg CO_2_eq); the GHGE for the low grain (6.5 CO_2_eq, 6.2, 6.9 CO_2_eq), restricted carbohydrate (6.7 CO_2_eq, 6.5, 6.9 CO_2_eq), and time restricted (6.1 CO_2_eq, 5.6, 6.7 CO_2_eq) diet patterns were similar; and the GHGE for the time restricted diet pattern was similar to the low fat diet pattern (5.8 CO_2_eq, 5.6, 6.7 CO_2_eq; Figure 1B). The GHGE for the general population was 6.1 kg CO_2_eq (5.9–6.2 kg CO_2_eq). The plant-based diet pattern had among the lowest diet cost ($13.79, 95% CI: $13.10, $14.47) and was similar to the time restricted ($15.98, 95% CI: $14.62, $17.33) diet pattern (Figure 1C). The low grain diet pattern ($18.45, $17.04, $19.85) was similar to the time restricted, low fat ($18.74, $17.90, $19.58), and restricted carbohydrate ($20.79, $20.06, $21.52) diet patterns. The diet cost of the general population was $18.87 ($18.42–$19.32).

### 3.4. Relationship of diet quality to greenhouse gas emissions and diet cost

**Figure 2:**
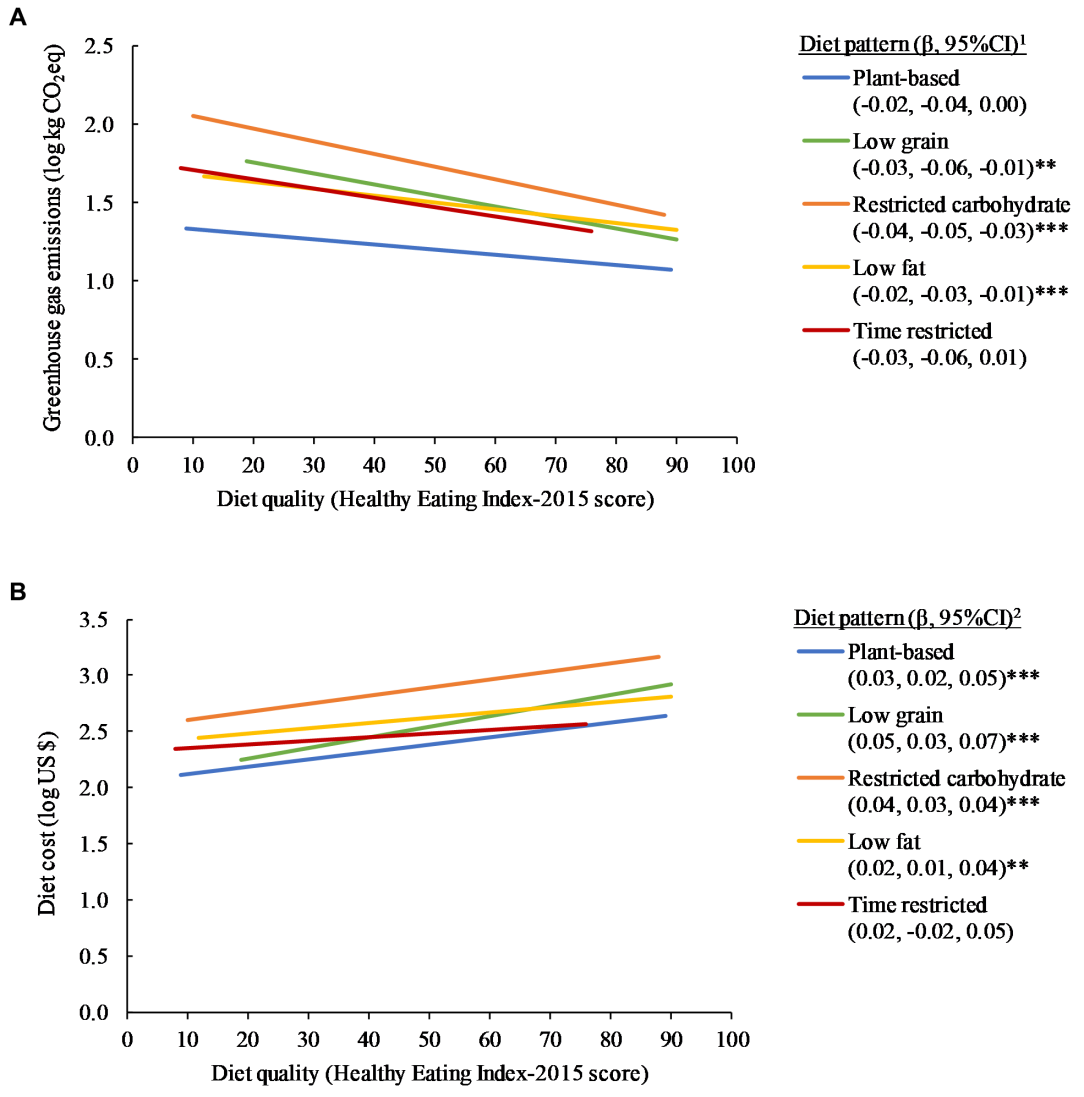
Relationship between daily diet quality **(A)** greenhouse gas emissions and **(B)** diet cost, for each popular diet pattern, 2011–2018 (*n* = 8,146). All results were adjusted for kcal and survey cycle using linear regression models. ^1^Change in log kg carbon dioxide equivalents (CO_2_eq) per 5-point increase in Healthy Eating Index-2015 score. ^2^Change in log food expenditure (US $) per 5-point increase in Healthy Eating Index-2015 score. Statistically different than *β* = 0 at *p* < 0.05 (*), *p* < 0.01 (**), and *p* < 0.001 (***), using Wald tests. General population (all adults categorized into a popular diet pattern and those not categorized and met all inclusion criteria, *n* = 18,969): β for log greenhouse gas emissions for 5 point increase in HEI-2015, −0.03 (95%CI: −0.03, −0.02), *p* < 0.001; log diet cost: 0.04 (95%CI: 0.04, 0.05), *p* < 0.001.

Greater diet quality was associated with lower GHGE (Figure 2A) for the low grain (*p* < 0.01), restricted carbohydrate (*p* < 0.001), and low-fat (*p* < 0.001) diet patterns, as well as for the general population (*p* < 0.001). Greater diet quality was not associated with GHGE for the plant-based and time restricted diet patterns (*p* > 0.05 for each diet pattern). Greater diet quality was associated with higher diet cost (Figure 2B) for the plant-based (*p* < 0.001), low grain (*p* < 0.001), restricted carbohydrate (*p* < 0.001), and low fat (*p* < 0.01) diet patterns, as well as for the general population (*p* < 0.001). Diet quality was not associated with diet cost for the time restricted diet pattern (*p* > 0.05). Higher diet cost was associated with higher GHGE (Supplementary Figure S2) for all diet patterns (*p* < 0.05 for all comparisons).

### 3.5. Food sources of greenhouse gas emissions and cost

**Figure 3:**
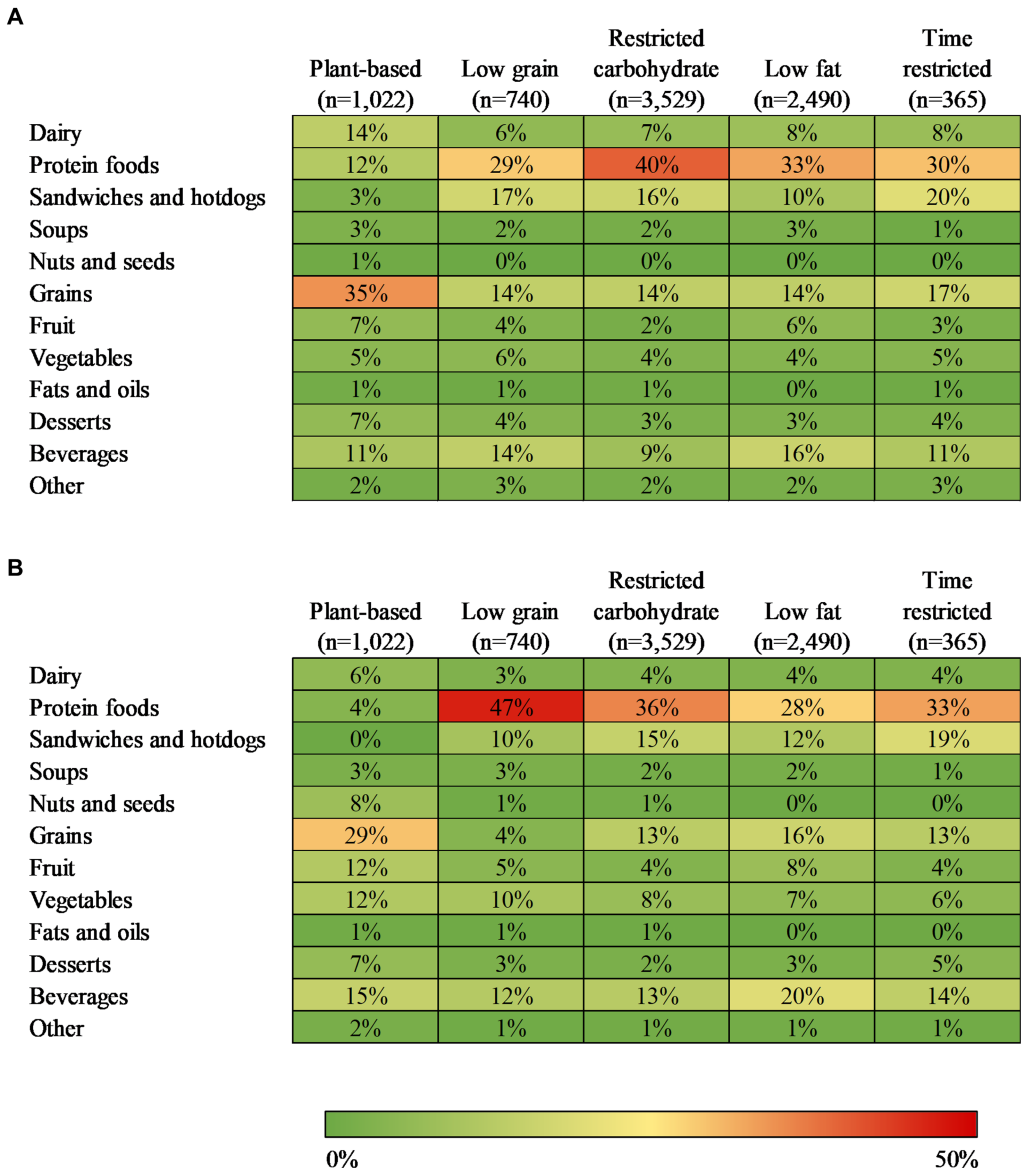
Contribution of food categories to mean daily **(A)** greenhouse gas emissions (kg CO_2_eq) and **(B)** diet cost (US $), for each popular diet pattern, 2011–2018 (*n* = 8,146). All results were adjusted for kcal and survey cycle using linear regression models. CO_2_eq, carbon dioxide equivalent. Diet categories represent predominant ingredient in mixed dish.

For all diet patterns except plant-based, protein foods accounted for the greatest share of GHGE (29–40%; Figure 3A), mostly from beef (Supplementary Tables S3–S7). This was followed by sandwiches and hotdogs (10–20%, mostly meat sandwiches, hotdogs, and sausages), grains (14–17%, mostly refined grain Mexican dishes, pasta, and pizza), and beverages (9–16%, mostly soft drinks with added sugar and alcohol). For the plant-based diet pattern, grains accounted for the greatest share of GHGE (35%, mostly from refined grains like Mexican dishes, pasta, and pizza), followed by dairy (14%, mostly fluid milk), protein foods (12%, mostly beef), and beverages (11%, mostly soft drinks with added sugar).

For all diet patterns except plant-based, protein foods accounted for the largest share (28–47%) of diet cost (Figure 3B), mostly from poultry and beef (Supplementary Tables S4–S7), followed by beverages (12–20%, mostly coffee and tea, soft drinks with added sugar, and alcohol). For the restricted carbohydrate, low fat, and time restricted diet patterns, sandwiches and hotdogs accounted for 12–19% of diet cost (mostly meat sandwiches, hotdogs, and sausages), followed by grains (13–16%, mostly refined grain Mexican dishes, breads, pizza, and rice). For the low grain diet pattern, sandwiches and hotdogs (mostly meat sandwiches, hotdogs, and sausages) and vegetables (mostly other vegetables) each accounted for 10% of diet cost. For the plant-based diet pattern (Supplementary Table S3), grains accounted for the largest share of diet cost (29%, mostly refined grain pizza, Mexican dishes, and breads), followed by beverages (15%, mostly coffee and tea), fruit (12%, mostly whole fruit without added sugar or fruit juice), and vegetables (12%, mostly other vegetables).

### 3.6. Sensitivity analyses

Eliminating the adjustment for losses and waste lowered GHGE by 35–37% for all diet patterns but the rank order remained the same (Supplementary Table S8). Removing the adjustment for losses and waste decreased diet cost for all diet patterns by 31–37% and increased statistical significance for most diet patterns but the rank order of diet patterns was similar (Supplementary Table S9). Eliminating the adjustment for FAFH prices further lowered diet cost by 19% for the plant-based diet pattern and by 32–37% for all other diet patterns. After eliminating the adjustment for losses, waste, and FAFH, the diet cost of the plant-based diet pattern ($7.04, 95% CI: $6.75, $7.33) was similar to the time restricted diet pattern ($6.90, 95% CI: $6.61, $7.19), the low grain diet pattern ($7.96, $7.47, $8.44) was similar to the low fat diet pattern ($8.64, $8.28, $9.01), and the low fat diet pattern was similar to the restricted carbohydrate diet pattern ($9.30, $8.52, $10.08).

Adjustment for demographic variables increased the mean HEI-2015 score of the time restricted diet pattern so that it was similar to the plant-based and restricted carbohydrate diet patterns but otherwise the rank order of diet patterns was unchanged; and including participants that were categorized into >1 diet pattern did not alter the rank order of diet patterns (Supplementary Table S10). Adjustment of mean GHGE using demographic variables did not modify the rank order of diet patterns, and including participants that were categorized into >1 diet pattern reduced the mean GHGE for all diet patterns and modified the statistical difference between some diet patterns but the rank order was similar (Supplementary Table S11). Adjustment of mean diet cost using demographic variables modified the statistical differences between diet patterns but the rank order was similar; and including participants that were categorized into >1 diet pattern reduced the mean diet cost for most diet patterns and increased statistical differences between diet patterns (Supplementary Table S12).

## 4. Discussion

In this nationally representative sample, higher diet quality was associated with lower GHGE and higher diet cost for most, but not all, popular diet patterns. Animal protein sources, mostly beef and poultry, contributed the largest share of GHGE and cost for most diet patterns. These findings can help inform major policy discussions in the US, which include identifying diet patterns that are healthy, environmentally sustainable, and affordable.

The present findings support prior observations that plant-based diet patterns have similar or higher mean diet quality (53) and lower mean GHGE (54) compared to average diets. Less is known about other diet patterns. Recent US-based studies have shown that plant-based diet patterns (vegetarian, vegan and pescatarian) had lower mean GHGE compared to keto and paleo diet patterns, but diet quality was similar across most diet patterns (20). Similarly, others have shown that plant-based diet patterns had lower GHGE compared to restricted carbohydrate, low grain, low fat, and time restricted diet patterns, but again diet quality was similar to most (21). The present study also supports prior findings that plant-based diet patterns were associated with lower costs both in the US (21) and globally (27). The present study is also consistent with reports that higher diet cost is associated with higher GHGE (55).

To our knowledge, only one other study (19) evaluated the relationship between the quality of plant-based diets and GHGE. In that study, higher diet quality was associated with lower GHGE (19), which is not consistent with the present results, showing no association between diet quality and GHGE among those participants who consumed plant-based diets. This discrepancy could be due to differences in the underlying study samples and dietary assessment methods. The Nurses’ Health Study II is based on a large sample of females and a food frequency questionnaire, whereas NHANES is based on a smaller but nationally representative sample of males and females and up to two 24 h dietary recalls. Further, GHGE data came from different LCA sources. The other study (19) used an index to measure adherence to a plant-based diet pattern on a continuous scale, whereas we limited the analysis to participants that consumed <1 ounce-equivalent of meat, poultry, and seafood. The present study also demonstrated that higher diet quality was associated with higher diet cost for all diet patterns except the time restricted diet pattern, which has not been shown previously.

No prior study has evaluated food sources of GHGE and cost in restricted carbohydrate, low grain, low fat, and time restricted diet patterns in the US. We show that protein foods, particularly beef, accounted for the largest share of GHGE, which is consistent with prior reports on national average diets in the US (12, 18, 24, 28, 29, 46, 55–58) and elsewhere (56). Modeling studies (24, 59) have shown that dairy accounted for the largest fraction (31–43%) of GHGE in healthy plant-based diet patterns, whereas the present study showed that refined grains accounted for the largest share of GHGE (35%), followed by dairy (14%). This difference is likely because other studies used modeled diet patterns that align with recommended intakes so they included greater amounts of dairy and lower amounts of refined grains than was documented in the present study, which evaluated actual plant-based diet patterns.

As consumers continue to adopt new diet patterns (16), we need a better understanding of their health impact, affordability, and environmental consequences to better guide public policy. Fortunately, several major federal efforts are underway in the US to provide an evidence base for future policy action. The USDA and Department of Health and Human Services (US HHS) have published proposed scientific questions for the 2025 Dietary Guidelines Advisory Committee (DGAC), which rely heavily on diet pattern analysis, and specifically ask for more evidence on the association between time restricted diet patterns and compliance with the Dietary Guidelines for Americans (DGA) (36). Although the 2025 DGAC will not specifically evaluate environmental sustainability and affordability (36), USDA and HHS have pledged to support other efforts to this end, including the establishment of a Federal Workgroup to assess the merits of including sustainability into future DGAs (15).

The National Strategy on Hunger, Nutrition, and Health proposes widespread efforts to support healthy, affordable, and environmentally friendly diet patterns (14). These include greater investment in research to understand whether new diet patterns should be developed and to evaluate the links between nutrition, environment, and affordability; improve data collection and metrics to inform policies on nutrition equity, access, and disparities; and increase collaboration between federal agencies to enhance data collection and sharing (14). The National Institutes of Health is also currently leading multiple Working Groups to characterize the evidence base that links diet patterns to other sustainability outcomes, to identify metrics and tools to measure these impacts and their interactions, and to identify knowledge gaps (15).

These federal efforts can be guided by three questions, which the present study can help answer. First, what are the sustainability trade-offs between diet patterns? Consistent (53) but not unanimous (20) evidence demonstrates that plant-based diet patterns are higher in diet quality and lower in GHGE than average diet patterns (54), yet these generalized comparisons obscure important nuances. These nuances depend on the type of plant-based diet (vegan versus lacto-ovo-vegetarian), the index used to measure diet quality, and the diet patterns being compared (20, 21). Furthermore, the present study shows that all popular diet patterns are low in diet quality (HEI-2015 scores between 44 and 53 out of 100), even though some are lower than others, which supports prior findings (17, 20, 21). In most cases, plant-based diet patterns have lower GHGE compared to other popular diet patterns and are lower in cost to some but not others. The restricted carbohydrate diet pattern is associated with higher GHGE and diet cost compared to all or most other popular diet patterns, but diet quality is similar to plant-based and low grain diet patterns. Other popular diet patterns have intermediate sustainability impacts or present trade-offs.

Second, what is the relationship between diet quality and other sustainability outcomes? Others have shown that greater adherence to the Dietary Approaches to Stop Hypertension (DASH) and Mediterranean diets are associated with lower GHGE among omnivores in the US, and that both of these indices correlate well with the HEI-2015 and Alternative Healthy Eating Index (AHEI-2010) (20). Others have shown that higher AHEI-2010 scores are associated with lower GHGE (19) and similar or lower use of agricultural resources like land, fertilizer nutrients, pesticides, and irrigation water among the general US population (19, 50), although others showed that higher HEI-2015 scores were associated with higher use of pesticides and irrigation water largely because of increased fruit and vegetable demands (50). Recently, others demonstrated that healthy plant-based diets were associated with lower GHGE and agricultural resource use, but diet quality wasn’t measured directly (19). To our knowledge, the present study is the first to evaluate the relationship of diet quality to GHGE and diet cost for a range of popular diet patterns in the US, which shows trade-offs between sustainability outcomes: higher diet quality was associated with lower GHGE and diet cost for restricted carbohydrate, low grain, and low fat diets; higher diet quality was associated with higher diet cost but no change in GHGE for plant-based diets; and higher diet quality was not associated with GHGE or diet cost for time-restricted diets.

Third, what are data gaps that need to be filled to improve diet sustainability analyses? NHANES provides a rich source of information on dietary intake that needs to be linked to data on environmental, economic, and social impacts, and the federal government is well poised to coordinate these efforts. Although others have developed methods for linking LCA data on GHGE and cumulative energy demand to NHANES foods, the largest fraction of these data represent agricultural-level impacts in European production systems (46); and more efforts are needed to expand this coverage to other sectors in other countries and to link NHANES foods with other environmental impacts such as biodiversity loss, soil erosion potential, water pollution, and others, and to make these data publicly available. Furthermore, these data linkages require FCID as a crosswalk, which has not been updated since 2010 (47). Methods for updating FCID to align with more recent NHANES data have been developed by others (48), and to link food commodities in FCID with loss and waste data from LAFA (51), but the federal government is in a better position to provide these services on an ongoing basis at regular intervals.

USDA provided data on food prices linked to foods in NHANES 2001–2004 but this effort was suspended for several decades, during which time researchers used the Consumer Price Index (CPI) to update food prices and imputation methods to link to more recent NHANES data (43). Fortunately, in 2023 USDA ERS released new price data linked to NHANES 2011–2018 foods (38), which were used for the first time in the present study. However, these data do not represent the price of FAFH, and researchers have developed methods for estimating these prices using FoodAPS (42), yet FoodAPS has not been released since 2013. The National Strategy on Hunger, Nutrition, and Health proposed another iteration of FoodAPS (14), which will facilitate diet sustainability analyses.

Several limitations of this study warrant mention. Dietary data were self-reported which may lead to inaccurate reporting by some participants due to social desirability bias. One day of dietary data were analyzed which does not represent usual intake, although it is appropriate for estimating *per capita* intake and comparing intake between groups (60). HEI-2015 was used to measure diet quality because it measures compliance with the DGA-2015 and is therefore relevant to ongoing policy discussions, but other diet quality indices are available and may produce different results (50). Most data on GHGE represented European agricultural systems and data from other regions and stages in the food system may produce different results. Finally, these findings do not account for changes in dietary behaviors or sustainability impacts that occurred after 2018 due to data availability limitations.

The present study also has several strengths. The multistage sampling design of NHANES and sampling weights make the dietary data generalizable to the US population, and sample sizes were large enough to establish mutually exclusive diet patterns. To fully account for the sustainability impacts of food choices, the environmental and economic impacts of losses and waste were included in addition to the impacts associated with the consumed portion. For the first time, this study utilized the 2011–2018 Purchase-to-Plate Price Tool [others have used the 2013–2016 version (21)], which represents scanner data from approximately 50% of all food retail sales in the US; and FAFH were adjusted to represent the higher price that consumers typically pay for foods purchased at food service establishments.

## 5. Conclusion

Higher diet quality is associated with lower GHGE for some, but not all, diet patterns, and is often accompanied by higher diet cost. These sustainability trade-offs can help inform major policy discussions in the US, which include developing and implementing programs to help consumers choose foods that maximize all domains of sustainability, and to encourage industry to support these efforts through sustainable production practices. This study also sheds light on further research areas needed to strengthen the evidence base for nutrition-sustainability interactions. The federal government is well poised to support such research efforts by standardizing data collection programs so they occur on a regular basis; coordinating data linkages, especially when these data are maintained by different federal agencies; and making these data publicly available to researchers.

## Data availability statement

Publicly available datasets were analyzed in this study. This data can be found at: https://www.cdc.gov/nchs/nhanes/about_nhanes.htm; https://www.ers.usda.gov/data-products/purchase-to-plate/; http://css.umich.edu/page/datafield.

## Ethics statement

The patients/participants provided their written informed consent to the National Center for Health Statistics to participate in the National Health and Nutrition Examination Survey. The present study is a secondary analysis and was reviewed and approved by the Institutional Review Board at William & Mary.

## Author contributions

ZC designed and conducted the research, was responsible for data management and analysis, and wrote the paper. ZC, AD, and DL read and edited the manuscript. All authors contributed to the article and approved the submitted version.

## Funding

This work was supported by the Commonwealth Center for Energy and the Environment at William & Mary, and the Summer Research Award provided by the Office of the Vice Provost at William & Mary. A portion of this work was supported by the Institute for the Advancement of Food and Nutrition Sciences (IAFNS) Carbohydrate Committee. The funders had no role in the design, implementation, analysis, or interpretation of the data.

## Conflict of interest

ZC has research awards from the Thomas F. and Kate Miller Jeffress Memorial Trust for a project unrelated to the present study; and received honoraria from the US Department of Agriculture, Routledge, MKYoung Food & Nutrition Strategies, National Geographic Society, The Ohio State University, and *Nutrition Today* for professional activities unrelated to the present research. AD is the original developer of the Naturally Nutrient Rich and the Nutrient Rich Food (NRF) indices. That work was supported at the time by the Nutrient Rich Coalition whose members were the Beef Checkoff Program through the National Cattlemen’s Beef Association, California Avocado Commission, California Kiwifruit, California Strawberry Commission, Egg Nutrition Center, Florida Department of Citrus, Grain Foods Foundation, National Dairy Council, National Pork Board, United States Potato Board, Wheat Foods Council, and Wild Blueberry Association of North America. AD is a member of scientific advisory boards for Nestlé and FrieslandCampina Institute and invited member of the Quality Carbohydrate Coalition supported by Potatoes USA. He has received multiple grants, contracts, and honoraria from entities both public and private with an interest in nutrient density metrics and nutrient profiling of foods.

The remaining author declares that the research was conducted in the absence of any commercial or financial relationships that could be construed as a potential conflict of interest.

## Publisher’s note

All claims expressed in this article are solely those of the authors and do not necessarily represent those of their affiliated organizations, or those of the publisher, the editors and the reviewers. Any product that may be evaluated in this article, or claim that may be made by its manufacturer, is not guaranteed or endorsed by the publisher.
