# Supplementary material for: Greater adherence to the Dietary Guidelines for Americans is associated with lower diet-related greenhouse gas emissions but higher costs
Source: Front Nutr. 2023 Aug 2;10:1220016. doi: 10.3389/fnut.2023.1220016 (PMC10433380; doi:10.3389/fnut.2023.1220016)
Supplement: Supplementary file 1 [file Data_Sheet_1.pdf]

Supplemental Table 1: Food and beverage categorization

Food and beverage category[1]	FNDDS code[2]	FPED condition[3]
Dairy		
Milk, fluid	111 and 115	
Milk, dry	118	
Cream	121	
Cheese	14 except 147	
Yogurt	114	
Milk and cream alternatives[4]	113 and 122	
Protein dishes		
Red meat		
Beef[5]	21 except 216; 2711, 2721, 2731, 2741, and 2811	
Pork[5,6]	22, 2712, 2722, 2732, 2742	
Lamb, goat, and veal[5,7]	23, 2713, 2723, 2733, 2743, and 2813	
Organ meat	251	
Poultry[5]	24, 2714, 2724, 2734, 2744, and 2814	
Seafood[5]	26 and 2715, 2725, 2735, 2745, 2815	
Eggs	31, 321, 324, and 35	
Plant proteins		
Meat alternatives[8]	418 and 59	
Egg alternatives[9]	33	
Beans, peas, and lentils	411, 412, and 413	
Sandwiches and hotdogs		
Hotdogs, sausages, and meat sandwiches[10]	252; 216; and 275 except 2755	
Seafood sandwiches	2755	
Egg sandwiches	322	
Nut butter sandwiches	423	
Soups		
Meat	283 except 2835 and 2836	
Seafood	2835	
Egg	323	

Cheese	147	
Plant-based soups		
Vegetable and legume	416, 718, 746, and 756	
Grain	584	
Nuts and seeds	411, 412, and 413	
Grain-based dishes		
Refined grain dishes		
Breads[11]	51 and 52	<50% whole grain (oz-equivalents)
Breakfast cereal	57	<50% whole grain (oz-equivalents)
Bars[12]	537 and 5354	<50% whole grain (oz-equivalents)
Salty snacks[13]	54	<50% whole grain (oz-equivalents)
Pancakes, waffles, and French toast[14]	55	<50% whole grain (oz-equivalents)
Pasta	561, 5813, and 5814	<50% whole grain (oz-equivalents)
Rice	562, 5815, and 5816	<50% whole grain (oz-equivalents)
Mexican dishes[15]	58100, 58101, 58102, 58103, 58104, and 58105	<50% whole grain (oz-equivalents)
Pizza[16]	58106, 58107, 58108, 58109, 5811, and 5812	<50% whole grain (oz-equivalents)
Whole grain dishes		
Breads[11]	51 and 52	≥50% whole grain (oz-equivalents)
Breakfast cereal	57	≥50% whole grain (oz-equivalents)
Bars[12]	537 and 5354	≥50% whole grain (oz-equivalents)
Salty snacks[13]	54	≥50% whole grain (oz-equivalents)
Pancakes, waffles, and French toast[14]	55	≥50% whole grain (oz-equivalents)
Pasta	561, 5813, and 5814	≥50% whole grain (oz-equivalents)
Rice	562, 5815, and 5816	≥50% whole grain (oz-equivalents)
Mexican dishes[15]	58100, 58101, 58102, 58103, 58104, and 58105	≥50% whole grain (oz-equivalents)
Pizza[16]	58106, 58107, 58108, 58109, 5811, and 5812	≥50% whole grain (oz-equivalents)
Fruit		
Whole		
No added sugar or fruit juice[17]	611; and 63 except 634	0 tspn added sugars and 0 cup-equivalents fruit juice
With added sugar or fruit juice[17]	611; and 63 except 634	>0 tspn added sugars or >0 cup-equivalents fruit juice

Dried	62	
Juice		
Juice, 100%[18]	612; 64 except 644; and 781	0 tspn added sugars
Juice, with added sugar[18]	612; 64 except 644; and 781	>0 tspn added sugars
Vegetables		
Dark green	72	
Red and orange	73, 741, 742, and 745	
Tomato juice	743	
Potatoes	71 except 718 and 719	
Other	75 except 756	
Fats and oils		
Table fats and spreads[19]	123 and 81	
Vegetable oils	82	
Salad dressings	83	
Desserts		
Cake and cookies[20]	53 except 537 and 5354	
Gelatin	915	
Ice cream[21]	131 and 132	
Non-dairy frozen[22]	916	
Fruit-based	634 except 63408010, 63408015, and 63409010	
Candy	917	
Beverages		
Coffee and tea	921, 922, and 923	
Soft drinks		
Soft drinks, with added sugar[23]	924	>0 tspn added sugars
Soft drinks, no added sugar	924 except 92410210 and 92410250	0 tspn added sugars
Carbonated water, no added sugar	92410210 and 92410250	
Fruit-flavored	925	
Energy	9265 and 9531	
Nutrition drinks and meal replacements	116, 951, 95201000, 95201010, 995201500, 95202, 9521, 9522, 9532, 9533, and 9534	
Water, non-carbonated	94	

Alcohol	93
Other[24]	All remaining foods and beverages

Adapted with permission from: Conrad et al. (2022). Quality of popular diet patterns in the United States: evaluating the effect of substitutions for foods high in added sugar, sodium, saturated fat, and refined grains. *Current Developments in Nutrition* , 12:nzac119.

[1]Predominant ingredient in mixed dish.

[2]Leading digits in each 8-digit food code from the USDA Food and Nutrient Database for Dietary Studies (FNDDS).

[3]Additional condition used to categorize foods, based on information provided by the USDA Food Patterns Equivalents Database (FPED).

[4]Plant-based milk alternatives including soy milk, rice milk, and coconut milk.

[5]Excludes sandwich meats.

[6]Includes bacon, spareribs, cracklings, skin, and miscellaneous parts.

[7]Includes game meat.

[8]Plant-based meat alternatives made from legumes and grains.

[9]Includes egg-free frozen mix, dry mix, and liquid mix.

[10]Includes deli meats and and meat spreads.

[11]Includes yeast breads and quick breads such as biscuits, muffins, cornbread, popovers, and tortillas.

[12]Includes granola bars, cereal bars, and nutrition bars.

[13]Includes crackers, chips, popcorn, and pretzels.

[14]Includes crepes and funnel cakes.

[15]Includes burritos, tacos, quesadillas, enchiladas, chimichangas, chalupas, gorditas, fajitas, tamales, and nachos.

[16]Includes dumplings, turnovers, fritters, and knishes.

[17]Includes canned and frozen fruit.

[18]Includes fruit and vegetable juice blends.

19Includes butter, margarine, and sour cream.

[20]Includes pies and pastries.

[21]Includes gelato, sherbet, pudding, and custard.

[22]Includes ices and popsicles.

[23]Includes carbonated water with added sugar.

[24]Includes condiments, icing, sauces, gravies, jams/jellies, sweeteners, non-specified meats, condensed/evaporated/powdered foods, and baby food.

Supplemental Table 2: Rationale for diet pattern categorization

Diet pattern	Rationale
Food group restricted	
Plant-based	Given the ubiquity of mixed dishes in the American diet, it is possible that some self-identified vegetarians and vegans unknowingly consume some amount of foods they avoid, even if this is not captured by dietary assessments. And in other cases, self-identified vegetarians have reported meat intake on dietary assessments.[1] Therefore, this diet pattern includes individuals that did not report any meat, poultry, or seafood in their dietary recall (n=406) as well as those who reported consuming <1 serving of meat, poultry, and seafood (8,147).
Low grain	Only 9 out of 8,147 participants in our sample consumed zero grains, which was insufficient to produce reliable nationally representative estimates. Therefore, this study used the 25th percentile of daily grain intake (4.42 ounce-equivalents) as the cutoff to define low grain intake.
Macronutrient restricted	
Restricted carbohydrate	Recommended daily carbohydrate intake for adults is 45-65% of kcal.[2] A restricted carbohydrate diet can be defined as <45% kcal from carbohydrates and includes moderate carbohydrate diets (26-44% kcal) and low carbohydrate diets (≤25% kcal).[3] The sample size for low-carbohydrate diets was insufficient to produce reliable nationally represenative estimates (n=114 out of 8,147) so we were not able to evaluate this group separately and therefore we combined them to represent restricted carbohydrate diets.
Low fat	To be consistent with other studies[3] we defined <30% kcal from fat as low intake.
Time restricted	Time restricted diets are commonly defined as kcal restriction for ≥12 consecutive hours per 24 hour period.[4] Of the 365 participants in our sample that restricted their kcal intake by ≥12 hours, 75% restricted by 12-14 hours, 17% restricted by 14-16 hours, and 8% restricted by >16 hours.

Adapted with permission from: Conrad et al. (2022). Quality of popular diet patterns in the United States: evaluating the effect of substitutions for foods high in added sugar, sodium, saturated fat, and refined grains. *Current Developments in Nutrition* , 12:nzac119.

[1]Parker and Vadiveloo. Diet quality of vegetarian diets compared with nonvegetarian diets: A systematic review. Nutr Rev 2019;77(3):144-60.

[2]Food and Nutrition Board, Institute of Medicine of the National Academies. (2005). Dietary Reference Intakes for energy, carbohydrate, fiber, fat, fatty acids, cholesterol, protein, and amino acids. The National Academies Press, Washington, DC.

[3]Kirkpatrick et al. (2019) Review of current evidence and clinical recommendations on the effects of low-carbohydrate and very-low-carbohydrate (including ketogenic) diets for the management of body weight and other cardiometabolic risk factors: A scientific statement from the National Lipid Association Nutrition and Lifestyle Task Force. *Journal of Clinical Lipidology* , 13:689-711.

[4]Freire. (2021). Scientific evidence of diets for weight loss: different macronutrient composition, intermittent fasting, and popular diets. *Nutrition* , 69:110549.

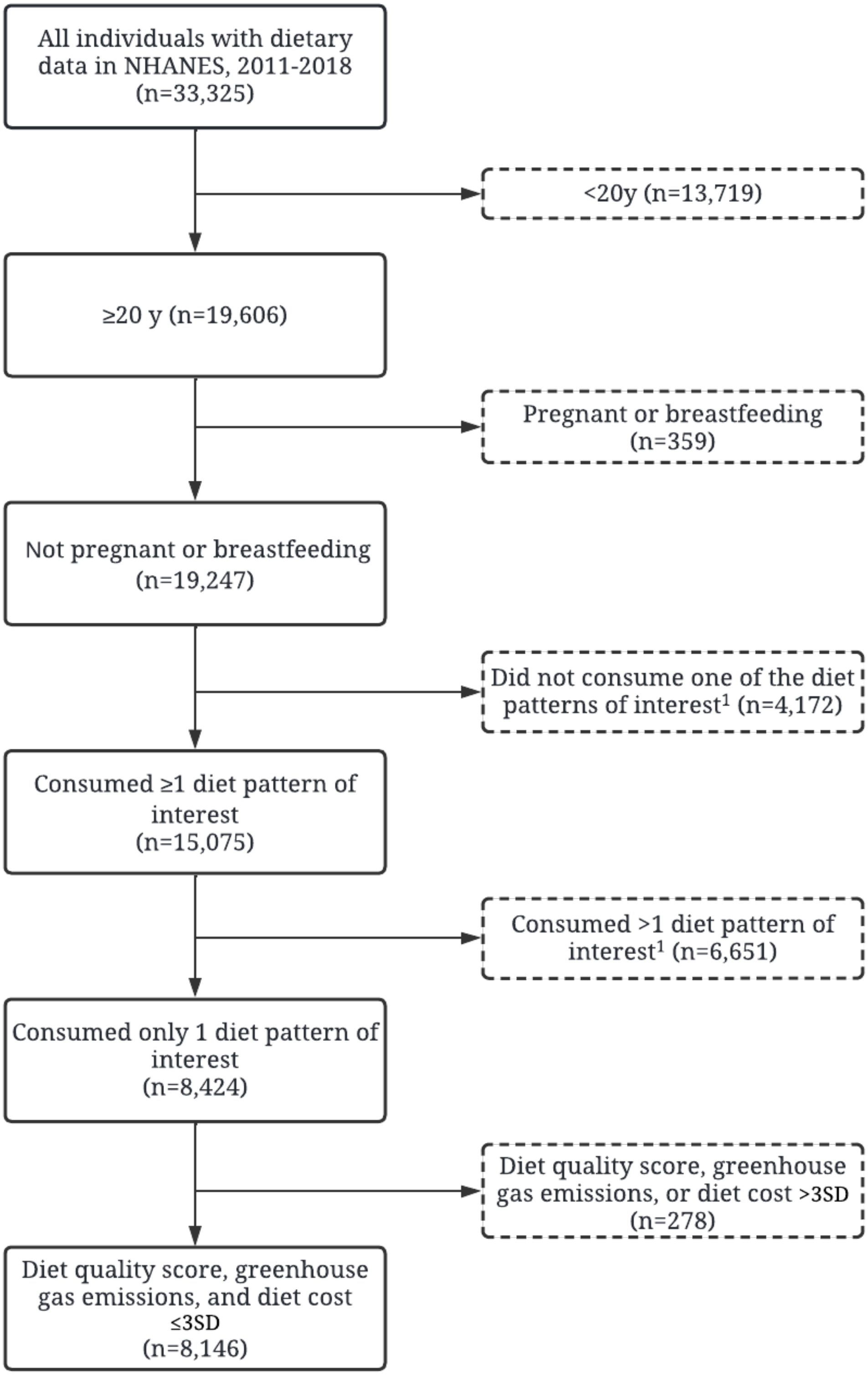

Supplemental Figure 1: Participant flowchart

NHANES, National Health and Nutrition Examination Survey

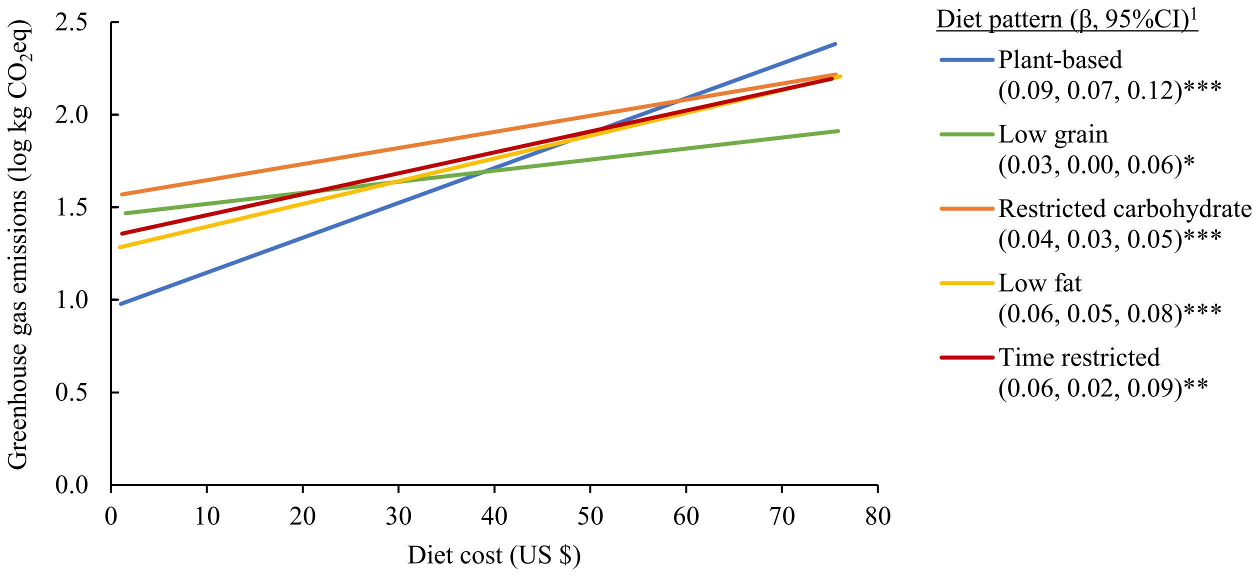

Supplemental Figure 2: Relationship between daily diet cost and greenhouse gas emissions, for each popular diet pattern, 2011-2018 (n=8,146)

All results were adjusted for kcal, and survey cycle using linear regression models.
[1]Change in log kg carbon dioxide equivalents (CO_2eq) per $1 increase in diet cost.
Statistically different than β=0 at $P<0.05$ (*), $P<0.01$ (**), and $P<0.001$ (***), using Wald tests.
General population (all adults categorized into a popular diet pattern and those not categorized and met all inclusion critiera, n=18,969): β for log greenhouse gas emissions for every $5 increase in diet cost, 0.04 (95%CI: 0.03, 0.05), $P<0.001$.

Supplemental Table 3: Mean daily greenhouse gas emissions and diet cost for the plant-based diet pattern, by food category, 2011-2018 (n=1,022)

Food and beverage category[1]	Greenhouse gas emissions (kg CO_2eq)	Cost (US $)
	Mean per day (95% CI)	
All foods	4.29 (4.03-4.55)	13.79 (13.1-14.47)
Dairy	0.57 (0.51-0.64)	0.98 (0.84-1.11)
Milk, fluid	0.29 (0.25-0.34)	0.31 (0.24-0.38)
Milk, dry	0.00 (0-0)	0.00 (0-0)
Cream	0.01 (0.01-0.01)	0.02 (0.01-0.03)
Cheese	0.19 (0.13-0.24)	0.38 (0.27-0.48)
Yogurt	0.07 (0.05-0.09)	0.21 (0.14-0.27)
Milk and cream alternatives[2]	0.02 (0.01-0.02)	0.06 (0.05-0.08)
Protein dishes	0.50 (0.37-0.63)	0.71 (0.55-0.86)
Red meat	0.28 (0.15-0.41)	0.13 (0.06-0.2)
Beef[3]	0.27 (0.14-0.4)	0.12 (0.05-0.19)
Pork[3,4]	0.01 (0-0.01)	0.01 (0-0.01)
Lamb, goat, and veal[3,5]	0.00 (0-0.01)	0.00 (0-0)
Organ meat	0.00 (0-0)	0.00 (0-0)
Poultry[3]	0.05 (0.02-0.07)	0.17 (0.06-0.27)
Seafood[3]	0.01 (-0.01-0.03)	0.00 (0-0)
Eggs	0.12 (0.1-0.15)	0.24 (0.16-0.33)
Plant proteins	0.04 (0.02-0.06)	0.17 (0.1-0.24)
Meat alternatives[6]	0.00 (0-0.01)	0.03 (0.01-0.05)
Egg alternatives[7]	0.00 (0-0)	0.00 (0-0)
Beans, peas, and lentils	0.03 (0.01-0.06)	0.14 (0.07-0.21)
Sandwiches and hotdogs	0.13 (0.06-0.19)	0.64 (0.33-0.96)
Hotdogs, sausages, and meat sandwiches[8]	0.11 (0.05-0.17)	0.51 (0.25-0.77)
Seafood sandwiches	0.00 (0-0)	0.00 (0-0)
Egg sandwiches	0.01 (0-0.02)	0.11 (0.01-0.21)
Nut butter sandwiches	0.01 (0-0.01)	0.03 (0.02-0.04)
Soups	0.13 (0.09-0.17)	0.39 (0.27-0.5)
Meat	0.02 (0.01-0.04)	0.09 (0.04-0.13)
Seafood	0.02 (0-0.03)	0.03 (0.01-0.05)
Egg	0.00 (0-0)	0.02 (-0.02-0.05)
Cheese	0.00 (0.05-0.13)	0.26 (0.16-0.35)
Plant-based soups	0.09 (0.05-0.13)	0.26 (0.16-0.35)
Vegetable and legume	0.06 (0.03-0.1)	0.17 (0.08-0.26)
Grain	0.02 (0.01-0.03)	0.08 (0.04-0.13)
Nuts and seeds	0.03 (0.02-0.04)	0.27 (0.18-0.37)
Grain-based dishes	1.41 (1.22-1.6)	4.46 (3.96-4.96)
Refined grain dishes	1.29 (1.1-1.48)	3.37 (3.01-3.74)
Breads[9]	0.06 (0.05-0.07)	0.63 (0.53-0.73)
Breakfast cereal	0.01 (0-0.01)	0.05 (0.03-0.07)
Bars[10]	0.01 (0-0.01)	0.05 (0.02-0.07)

Salty snacks[11]	0.02 (0.01-0.02)	0.20 (0.16-0.24)
Pancakes, waffles, and French toast[12]	0.01 (0-0.01)	0.04 (0.02-0.06)
Pasta	0.34 (0.22-0.46)	0.50 (0.34-0.66)
Rice	0.05 (0-0.1)	0.16 (0.07-0.25)
Mexican dishes[13]	0.46 (0.27-0.65)	0.74 (0.48-0.99)
Pizza[14]	0.34 (0.27-0.42)	1.01 (0.77-1.25)
Whole grain dishes	0.12 (0.09-0.15)	1.08 (0.75-1.42)
Breads[9]	0.01 (0.01-0.02)	0.14 (0.11-0.17)
Breakfast cereal	0.01 (0.01-0.02)	0.11 (0.08-0.14)
Bars[10]	0.01 (0-0.01)	0.06 (0.02-0.11)
Salty snacks[11]	0.06 (0.03-0.09)	0.70 (0.38-1.02)
Pancakes, waffles, and French toast[12]	0.00 (0-0)	0.00 (0-0)
Pasta	0.01 (0-0.03)	0.01 (0-0.02)
Rice	0.01 (0.01-0.02)	0.06 (0.04-0.08)
Mexican dishes[13]	0.00 (0-0.01)	0.00 (0-0)
Pizza[14]	0.00 (0-0)	0.00 (0-0)
Fruit	0.27 (0.2-0.34)	1.54 (1.17-1.92)
Whole	0.19 (0.12-0.26)	1.31 (0.95-1.67)
No added sugar or fruit juice[15]	0.18 (0.12-0.25)	1.27 (0.92-1.62)
With added sugar or fruit juice[15]	0.00 (0-0.01)	0.04 (0.01-0.07)
Dried	0.00 (0-0.01)	0.03 (0.01-0.05)
Juice	0.08 (0.05-0.11)	0.20 (0.1-0.29)
Juice, 100%[16]	0.07 (0.05-0.1)	0.17 (0.12-0.22)
Juice, with added sugar[16]	0.00 (0-0.01)	0.03 (-0.02-0.09)
Vegetables	0.22 (0.18-0.26)	1.64 (1.29-1.98)
Dark green	0.02 (0.01-0.04)	0.25 (0.12-0.38)
Red and orange	0.01 (0.01-0.02)	0.13 (0.1-0.15)
Tomato juice	0.00 (0-0)	0.01 (0-0.02)
Potatoes	0.03 (0.01-0.05)	0.14 (0.12-0.17)
Other	0.15 (0.11-0.19)	1.11 (0.83-1.39)
Fats and oils	0.05 (0.04-0.07)	0.09 (0.07-0.11)
Table fats and spreads[17]	0.04 (0.03-0.05)	0.05 (0.03-0.06)
Vegetable oils	0.00 (0-0)	0.00 (0-0)
Salad dressings	0.01 (0.01-0.02)	0.05 (0.03-0.06)
Desserts	0.27 (0.21-0.33)	1.16 (0.82-1.49)
Cake and cookies[18]	0.13 (0.08-0.18)	0.78 (0.47-1.1)
Gelatin	0.00 (0-0)	0.00 (0-0)
Ice cream[19]	0.10 (0.07-0.12)	0.15 (0.11-0.19)
Non-dairy frozen[20]	0.00 (0-0)	0.00 (0-0)
Fruit-based	0.00 (0-0)	0.05 (0-0.1)
Candy	0.04 (0.03-0.05)	0.17 (0.13-0.21)
Beverages	0.45 (0.39-0.51)	0.99 (0.9-1.09)
Coffee and tea	0.09 (0.07-0.11)	0.33 (0.27-0.4)

Soft drinks	0.21 (0.16-0.26)	0.21 (0.18-0.25)
Soft drinks, with added sugar[21]	0.21 (0.16-0.26)	0.14 (0.11-0.18)
Soft drinks, no added sugar	0.00 (0-0)	0.07 (0.03-0.1)
Fruit-flavored	0.04 (0.03-0.06)	0.03 (0.02-0.04)
Energy	0.01 (0-0.01)	0.02 (0.01-0.02)
Nutrition drinks and meal replacements	0.03 (0.02-0.05)	0.03 (0.02-0.05)
Water, non-carbonated	0.02 (0.01-0.03)	0.19 (0.16-0.22)
Alcohol	0.04 (0.03-0.06)	0.16 (0.11-0.22)
Other[22]	0.07 (0.04-0.1)	0.30 (0.22-0.39)

All results were adjusted for kcal and survey cycle using linear regression models.

CO_2eq, carbon dioxide equivalent

[1]Predominant ingredient in mixed dish.

[2]Plant-based milk alternatives including soy milk, rice milk, and coconut milk.

[3]Excludes sandwich meats.

[4]Includes bacon, spareribs, cracklings, skin, and miscellaneous parts.

[5]Includes game meat.

[6]Plant-based meat alternatives made from legumes and grains.

[7]Includes egg-free frozen mix, dry mix, and liquid mix.

[8]Includes deli meats and and meat spreads.

[9]Includes yeast breads and quick breads such as biscuits, muffins, cornbread, popovers, and tortillas.

[10]Includes granola bars, cereal bars, and nutrition bars.

[11]Includes crackers, chips, popcorn, and pretzels.

[12]Includes crepes and funnel cakes.

[13]Includes burritos, tacos, quesadillas, enchiladas, chimichangas, chalupas, gorditas, fajitas, tamales, and

[14]Includes dumplings, turnovers, fritters, and knishes.

[15]Includes canned and frozen fruit.

[16]Includes fruit and vegetable juice blends.

[17]Includes butter, margarine, and sour cream.

[18]Includes pies and pastries.

[19]Includes gelato, sherbet, pudding, and custard.

[20]Includes ices and popsicles.

[21]Includes carbonated water with added sugar.

[22]Includes condiments, icing, sauces, gravies, jams/jellies, sweeteners, non-specified meats,

Supplemental Table 4: Mean daily greenhouse gas emissions and diet cost for the low grain diet pattern, by food category, 2011-2018 (n=740)

Food and beverage category[1]	Greenhouse gas emissions (kg CO_2eq)	Cost (US $)
	Mean per day (95% CI)	
All foods	6.54 (6.22-6.87)	18.45 (17.04-19.85)
Dairy	0.38 (0.32-0.44)	0.71 (0.54-0.87)
Milk, fluid	0.20 (0.16-0.23)	0.24 (0.18-0.29)
Milk, dry	0.00 (0-0)	0.00 (0-0)
Cream	0.01 (0.01-0.02)	0.02 (0.01-0.03)
Cheese	0.10 (0.08-0.13)	0.23 (0.17-0.28)
Yogurt	0.04 (0.02-0.07)	0.16 (0.05-0.26)
Milk and cream alternatives[2]	0.02 (0.01-0.02)	0.07 (0.04-0.09)
Protein dishes	1.70 (1.46-1.94)	4.75 (3.73-5.77)
Red meat	1.10 (0.87-1.34)	1.76 (1.31-2.22)
Beef[3]	0.93 (0.7-1.15)	1.04 (0.65-1.44)
Pork[3,4]	0.15 (0.11-0.19)	0.70 (0.43-0.97)
Lamb, goat, and veal[3,5]	0.03 (0-0.07)	0.02 (-0.01-0.05)
Organ meat	0.00 (0-0)	0.00 (0-0)
Poultry[3]	0.29 (0.23-0.35)	1.73 (1.05-2.41)
Seafood[3]	0.19 (0.11-0.26)	0.74 (0.46-1.02)
Eggs	0.10 (0.07-0.14)	0.48 (0.2-0.76)
Plant proteins	0.01 (0-0.03)	0.04 (0.02-0.06)
Meat alternatives[6]	0.00 (0-0)	0.00 (0-0)
Egg alternatives[7]	0.00 (0-0)	0.00 (0-0)
Beans, peas, and lentils	0.01 (0-0.03)	0.04 (0.02-0.06)
Sandwiches and hotdogs	1.03 (0.82-1.24)	3.30 (2.22-4.37)
Hotdogs, sausages, and meat sandwiches[8]	1.01 (0.8-1.21)	3.13 (2.06-4.2)
Seafood sandwiches	0.00 (0-0.01)	0.06 (0.01-0.11)
Egg sandwiches	0.01 (0-0.03)	0.09 (-0.03-0.21)
Nut butter sandwiches	0.00 (0-0.01)	0.01 (0-0.03)
Soups	0.11 (0.03-0.18)	0.25 (0.15-0.34)
Meat	0.06 (0-0.13)	0.14 (0.06-0.22)
Seafood	0.01 (0-0.01)	0.02 (-0.01-0.04)
Egg	0.00 (0-0)	0.02 (-0.02-0.06)
Cheese	0.00 (0-0)	0.00 (0-0)
Plant-based soups	0.04 (0-0.08)	0.07 (0.03-0.1)
Vegetable and legume	0.01 (0-0.03)	0.04 (0.01-0.08)
Grain	0.02 (-0.01-0.06)	0.03 (0.01-0.04)
Nuts and seeds	0.01 (0.01-0.01)	0.13 (0.02-0.25)
Grain-based dishes	0.86 (0.65-1.06)	1.84 (1.46-2.21)
Refined grain dishes	0.80 (0.6-1.01)	1.60 (1.26-1.94)
Breads[9]	0.02 (0.01-0.02)	0.16 (0.11-0.2)
Breakfast cereal	0.00 (0-0)	0.02 (0.01-0.03)

Bars[10]	0.00 (0-0)	0.01 (0-0.02)
Salty snacks[11]	0.00 (0-0)	0.00 (0-0.01)
Pancakes, waffles, and French toast[12]	0.01 (0-0.01)	0.03 (0.01-0.05)
Pasta	0.29 (0.15-0.42)	0.41 (0.24-0.57)
Rice	0.04 (0.03-0.06)	0.30 (0.13-0.47)
Mexican dishes[13]	0.35 (0.21-0.49)	0.37 (0.24-0.49)
Pizza[14]	0.09 (0.04-0.15)	0.31 (0.07-0.55)
Whole grain dishes	0.06 (0.02-0.1)	0.24 (0.11-0.36)
Breads[9]	0.00 (0-0)	0.01 (0-0.02)
Breakfast cereal	0.00 (0-0.01)	0.04 (0.02-0.06)
Bars[10]	0.01 (0-0.02)	0.04 (-0.01-0.1)
Salty snacks[11]	0.01 (0-0.01)	0.08 (-0.02-0.18)
Pancakes, waffles, and French toast[12]	0.00 (0-0)	0.00 (0-0.01)
Pasta	0.01 (-0.01-0.02)	0.00 (0-0.01)
Rice	0.01 (0.01-0.02)	0.06 (0.03-0.09)
Mexican dishes[13]	0.02 (-0.02-0.05)	0.00 (0-0)
Pizza[14]	0.00 (0-0)	0.00 (0-0)
Fruit	0.23 (0.18-0.28)	1.29 (1.05-1.53)
Whole	0.14 (0.1-0.18)	1.04 (0.84-1.24)
No added sugar or fruit juice[15]	0.13 (0.09-0.17)	0.98 (0.79-1.18)
With added sugar or fruit juice[15]	0.01 (0-0.02)	0.06 (0-0.11)
Dried	0.00 (0-0.01)	0.03 (0.01-0.05)
Juice	0.09 (0.07-0.12)	0.22 (0.13-0.31)
Juice, 100%[16]	0.09 (0.06-0.11)	0.21 (0.12-0.29)
Juice, with added sugar[16]	0.00 (0-0.01)	0.01 (-0.01-0.03)
Vegetables	0.34 (0.26-0.42)	2.02 (1.69-2.35)
Dark green	0.01 (0.01-0.02)	0.15 (0.09-0.2)
Red and orange	0.02 (0.01-0.02)	0.21 (0.12-0.3)
Tomato juice	0.00 (0-0)	0.00 (0-0.01)
Potatoes	0.09 (0.04-0.13)	0.42 (0.36-0.48)
Other	0.22 (0.16-0.28)	1.24 (0.95-1.52)
Fats and oils	0.03 (0.02-0.04)	0.07 (0.05-0.09)
Table fats and spreads[17]	0.01 (0.01-0.02)	0.02 (0.01-0.03)
Vegetable oils	0.00 (0-0)	0.00 (0-0)
Salad dressings	0.02 (0.01-0.03)	0.05 (0.03-0.07)
Desserts	0.25 (0.2-0.3)	0.85 (0.7-1)
Cake and cookies[18]	0.06 (0.04-0.07)	0.35 (0.24-0.47)
Gelatin	0.00 (0-0.01)	0.00 (0-0.01)
Ice cream[19]	0.15 (0.1-0.19)	0.25 (0.17-0.33)
Non-dairy frozen[20]	0.00 (0-0)	0.01 (0-0.01)
Fruit-based	0.00 (0-0)	0.02 (-0.02-0.06)
Candy	0.04 (0.02-0.06)	0.21 (0.13-0.29)
Beverages	0.85 (0.72-0.99)	1.33 (1.13-1.53)

Coffee and tea	0.10 (0.07-0.14)	0.43 (0.28-0.59)
Soft drinks	0.48 (0.37-0.59)	0.34 (0.29-0.39)
Soft drinks, with added sugar[21]	0.48 (0.37-0.59)	0.30 (0.25-0.36)
Soft drinks, no added sugar	0.00 (0-0)	0.04 (0.02-0.05)
Fruit-flavored	0.08 (0.04-0.12)	0.07 (0.04-0.09)
Energy	0.02 (0.01-0.03)	0.06 (0.01-0.1)
Nutrition drinks and meal replacements	0.12 (0.05-0.19)	0.11 (0.03-0.18)
Water, non-carbonated	0.03 (0-0.06)	0.21 (0.18-0.24)
Alcohol	0.03 (0.01-0.04)	0.11 (0.05-0.17)
Other[22]	0.17 (0.1-0.25)	0.34 (0.21-0.47)

All results were adjusted for kcal and survey cycle using linear regression models.

CO_2eq, carbon dioxide equivalent

[1]Predominant ingredient in mixed dish.

[2]Plant-based milk alternatives including soy milk, rice milk, and coconut milk.

[3]Excludes sandwich meats.

[4]Includes bacon, spareribs, cracklings, skin, and miscellaneous parts.

[5]Includes game meat.

[6]Plant-based meat alternatives made from legumes and grains.

[7]Includes egg-free frozen mix, dry mix, and liquid mix.

[8]Includes deli meats and and meat spreads.

[9]Includes yeast breads and quick breads such as biscuits, muffins, cornbread, popovers, and tortillas.

[10]Includes granola bars, cereal bars, and nutrition bars.

[11]Includes crackers, chips, popcorn, and pretzels.

[12]Includes crepes and funnel cakes.

[13]Includes burritos, tacos, quesadillas, enchiladas, chimichangas, chalupas, gorditas, fajitas, tamales, and

[14]Includes dumplings, turnovers, fritters, and knishes.

[15]Includes canned and frozen fruit.

[16]Includes fruit and vegetable juice blends.

[17]Includes butter, margarine, and sour cream.

[18]Includes pies and pastries.

[19]Includes gelato, sherbet, pudding, and custard.

[20]Includes ices and popsicles.

[21]Includes carbonated water with added sugar.

[22]Includes condiments, icing, sauces, gravies, jams/jellies, sweeteners, non-specified meats,

Supplemental Table 5: Mean daily greenhouse gas emissions and diet cost for the restricted carbohydrate diet pattern, by food category, 2011-2018 (n=3,529)

Food and beverage category[1]	Greenhouse gas emissions (kg CO_2eq)	Cost (US $)
	Mean per day (95% CI)	
All foods	6.68 (6.22-6.87)	20.79 (20.06-21.52)
Dairy	0.51 (0.48-0.55)	1.01 (0.92-1.1)
Milk, fluid	0.20 (0.18-0.23)	0.25 (0.18-0.32)
Milk, dry	0.00 (0-0)	0.00 (0-0)
Cream	0.02 (0.01-0.02)	0.03 (0.02-0.04)
Cheese	0.24 (0.21-0.27)	0.54 (0.49-0.59)
Yogurt	0.04 (0.03-0.05)	0.13 (0.1-0.16)
Milk and cream alternatives[2]	0.01 (0.01-0.02)	0.05 (0.04-0.07)
Protein dishes	2.81 (2.65-2.97)	7.04 (6.59-7.49)
Red meat	1.94 (1.78-2.1)	2.96 (2.6-3.32)
Beef[3]	1.67 (1.51-1.83)	1.88 (1.57-2.18)
Pork[3,4]	0.18 (0.16-0.2)	0.98 (0.79-1.17)
Lamb, goat, and veal[3,5]	0.06 (0.03-0.1)	0.07 (0.03-0.1)
Organ meat	0.03 (-0.01-0.06)	0.04 (-0.01-0.08)
Poultry[3]	0.35 (0.33-0.38)	1.95 (1.75-2.15)
Seafood[3]	0.28 (0.22-0.34)	1.07 (0.93-1.22)
Eggs	0.22 (0.2-0.24)	0.97 (0.81-1.13)
Plant proteins	0.02 (0.01-0.02)	0.09 (0.07-0.11)
Meat alternatives[6]	0.00 (0-0)	0.01 (0-0.02)
Egg alternatives[7]	0.00 (0-0.01)	0.01 (0-0.01)
Beans, peas, and lentils	0.01 (0.01-0.01)	0.07 (0.05-0.09)
Sandwiches and hotdogs	1.16 (1.08-1.24)	3.42 (2.97-3.87)
Hotdogs, sausages, and meat sandwiches[8]	1.14 (1.06-1.22)	3.08 (2.67-3.5)
Seafood sandwiches	0.01 (0-0.01)	0.13 (0.04-0.23)
Egg sandwiches	0.02 (0.01-0.03)	0.20 (0.12-0.28)
Nut butter sandwiches	0.00 (0-0)	0.01 (0.01-0.01)
Soups	0.13 (0.09-0.16)	0.35 (0.26-0.44)
Meat	0.03 (0.02-0.04)	0.12 (0.08-0.16)
Seafood	0.02 (0-0.04)	0.07 (0.01-0.14)
Egg	0.00 (0-0)	0.02 (-0.02-0.06)
Cheese	0.00 (0-0)	0.00 (0-0)
Plant-based soups	0.07 (0.04-0.1)	0.14 (0.09-0.18)
Vegetable and legume	0.06 (0.03-0.09)	0.09 (0.06-0.13)
Grain	0.01 (0.01-0.02)	0.04 (0.02-0.06)
Nuts and seeds	0.03 (0.03-0.04)	0.31 (0.25-0.36)
Grain-based dishes	1.01 (0.89-1.12)	3.37 (3.18-3.56)
Refined grain dishes	0.92 (0.8-1.03)	2.43 (2.29-2.57)
Breads[9]	0.05 (0.05-0.06)	0.55 (0.51-0.6)
Breakfast cereal	0.00 (0-0)	0.02 (0.01-0.02)
Bars[10]	0.00 (0-0.01)	0.03 (0.02-0.04)

Salty snacks[11]	0.01 (0.01-0.02)	0.19 (0.16-0.23)
Pancakes, waffles, and French toast[12]	0.01 (0.01-0.01)	0.05 (0.03-0.06)
Pasta	0.11 (0.08-0.15)	0.27 (0.21-0.33)
Rice	0.04 (0.03-0.06)	0.20 (0.15-0.24)
Mexican dishes[13]	0.52 (0.41-0.63)	0.67 (0.55-0.79)
Pizza[14]	0.16 (0.14-0.18)	0.46 (0.39-0.53)
Whole grain dishes	0.09 (0.07-0.1)	0.94 (0.79-1.09)
Breads[9]	0.01 (0.01-0.01)	0.10 (0.07-0.12)
Breakfast cereal	0.01 (0-0.01)	0.05 (0.04-0.07)
Bars[10]	0.01 (0-0.01)	0.06 (0.04-0.08)
Salty snacks[11]	0.06 (0.04-0.07)	0.68 (0.54-0.82)
Pancakes, waffles, and French toast[12]	0.00 (0-0)	0.00 (0-0)
Pasta	0.00 (0-0.01)	0.00 (0-0)
Rice	0.01 (0.01-0.01)	0.04 (0.03-0.05)
Mexican dishes[13]	0.00 (0-0)	0.00 (0-0)
Pizza[14]	0.00 (0-0)	0.00 (0-0.01)
Fruit	0.13 (0.12-0.15)	0.89 (0.79-0.99)
Whole	0.09 (0.08-0.1)	0.76 (0.66-0.86)
No added sugar or fruit juice[15]	0.08 (0.07-0.09)	0.71 (0.62-0.81)
With added sugar or fruit juice[15]	0.01 (0-0.01)	0.05 (0.03-0.06)
Dried	0.00 (0-0)	0.02 (0.01-0.03)
Juice	0.04 (0.03-0.05)	0.11 (0.09-0.13)
Juice, 100%[16]	0.04 (0.03-0.05)	0.10 (0.08-0.12)
Juice, with added sugar[16]	0.00 (0-0)	0.01 (0-0.02)
Vegetables	0.26 (0.24-0.27)	1.97 (1.84-2.1)
Dark green	0.02 (0.01-0.02)	0.23 (0.19-0.26)
Red and orange	0.02 (0.01-0.02)	0.20 (0.18-0.23)
Tomato juice	0.00 (0-0)	0.01 (0-0.01)
Potatoes	0.06 (0.05-0.07)	0.38 (0.35-0.42)
Other	0.16 (0.14-0.18)	1.15 (1.04-1.26)
Fats and oils	0.07 (0.07-0.08)	0.17 (0.15-0.18)
Table fats and spreads[17]	0.04 (0.04-0.05)	0.06 (0.05-0.08)
Vegetable oils	0.00 (0-0)	0.01 (0-0.01)
Salad dressings	0.03 (0.02-0.03)	0.10 (0.08-0.11)
Desserts	0.20 (0.18-0.22)	0.85 (0.75-0.95)
Cake and cookies[18]	0.08 (0.07-0.09)	0.52 (0.43-0.61)
Gelatin	0.00 (0-0)	0.00 (0-0.01)
Ice cream[19]	0.08 (0.07-0.09)	0.16 (0.14-0.19)
Non-dairy frozen[20]	0.00 (0-0)	0.00 (0-0)
Fruit-based	0.00 (0-0)	0.02 (0.01-0.03)
Candy	0.03 (0.03-0.04)	0.14 (0.11-0.17)
Beverages	0.61 (0.56-0.65)	2.30 (1.98-2.62)
Coffee and tea	0.10 (0.08-0.11)	0.56 (0.27-0.85)

Soft drinks	0.11 (0.09-0.13)	0.17 (0.16-0.19)
Soft drinks, with added sugar[21]	0.11 (0.09-0.13)	0.08 (0.07-0.09)
Soft drinks, no added sugar	0.00 (0-0)	0.10 (0.08-0.11)
Fruit-flavored	0.03 (0.03-0.04)	0.03 (0.02-0.04)
Energy	0.01 (0-0.02)	0.05 (0.01-0.09)
Nutrition drinks and meal replacements	0.06 (0.03-0.09)	0.05 (0.03-0.07)
Water, non-carbonated	0.02 (0.01-0.03)	0.23 (0.2-0.25)
Alcohol	0.27 (0.23-0.31)	1.18 (1-1.36)
Other[22]	0.13 (0.1-0.16)	0.35 (0.3-0.4)

All results were adjusted for kcal and survey cycle using linear regression models.

CO_2eq, carbon dioxide equivalent

[1]Predominant ingredient in mixed dish.

[2]Plant-based milk alternatives including soy milk, rice milk, and coconut milk.

[3]Excludes sandwich meats.

[4]Includes bacon, spareribs, cracklings, skin, and miscellaneous parts.

[5]Includes game meat.

[6]Plant-based meat alternatives made from legumes and grains.

[7]Includes egg-free frozen mix, dry mix, and liquid mix.

[8]Includes deli meats and and meat spreads.

[9]Includes yeast breads and quick breads such as biscuits, muffins, cornbread, popovers, and tortillas.

[10]Includes granola bars, cereal bars, and nutrition bars.

[11]Includes crackers, chips, popcorn, and pretzels.

[12]Includes crepes and funnel cakes.

[13]Includes burritos, tacos, quesadillas, enchiladas, chimichangas, chalupas, gorditas, fajitas, tamales, and

[14]Includes dumplings, turnovers, fritters, and knishes.

[15]Includes canned and frozen fruit.

[16]Includes fruit and vegetable juice blends.

[17]Includes butter, margarine, and sour cream.

[18]Includes pies and pastries.

[19]Includes gelato, sherbet, pudding, and custard.

[20]Includes ices and popsicles.

[21]Includes carbonated water with added sugar.

[22]Includes condiments, icing, sauces, gravies, jams/jellies, sweeteners, non-specified meats,

Supplemental Table 6: Mean daily greenhouse gas emissions and diet cost for the low fat diet pattern, by food category, 2011-2018 (n=2,490)

Food and beverage category[1]	Greenhouse gas emissions (kg CO_2eq)	Cost (US $)
	Mean per day (95% CI)	
All foods	5.83 (5.61-6.04)	18.74 (17.9-19.58)
Dairy	0.46 (0.42-0.51)	0.84 (0.75-0.93)
Milk, fluid	0.26 (0.23-0.29)	0.30 (0.25-0.36)
Milk, dry	0.00 (0-0)	0.00 (0-0)
Cream	0.01 (0-0.01)	0.01 (0-0.02)
Cheese	0.11 (0.09-0.13)	0.24 (0.2-0.28)
Yogurt	0.08 (0.06-0.09)	0.24 (0.18-0.3)
Milk and cream alternatives[2]	0.01 (0.01-0.01)	0.05 (0.04-0.06)
Protein dishes	1.80 (1.62-1.97)	4.40 (4-4.8)
Red meat	1.25 (1.08-1.42)	1.99 (1.67-2.31)
Beef[3]	1.04 (0.88-1.21)	1.22 (0.93-1.51)
Pork[3,4]	0.16 (0.12-0.19)	0.70 (0.53-0.88)
Lamb, goat, and veal[3,5]	0.05 (0.01-0.08)	0.07 (0.01-0.12)
Organ meat	0.01 (0-0.01)	0.00 (0-0.01)
Poultry[3]	0.24 (0.21-0.27)	1.20 (0.96-1.44)
Seafood[3]	0.22 (0.16-0.28)	0.95 (0.7-1.21)
Eggs	0.06 (0.05-0.07)	0.16 (0.11-0.21)
Plant proteins	0.02 (0.02-0.03)	0.10 (0.08-0.12)
Meat alternatives[6]	0.00 (0-0)	0.01 (0-0.03)
Egg alternatives[7]	0.00 (0-0.01)	0.00 (0-0)
Beans, peas, and lentils	0.02 (0.01-0.02)	0.08 (0.07-0.1)
Sandwiches and hotdogs	0.58 (0.48-0.67)	2.21 (1.73-2.69)
Hotdogs, sausages, and meat sandwiches[8]	0.56 (0.47-0.66)	2.09 (1.66-2.53)
Seafood sandwiches	0.00 (0-0)	0.03 (0.01-0.05)
Egg sandwiches	0.01 (0-0.01)	0.08 (-0.02-0.18)
Nut butter sandwiches	0.00 (0-0)	0.01 (0-0.02)
Soups	0.14 (0.11-0.18)	0.32 (0.25-0.39)
Meat	0.06 (0.04-0.09)	0.13 (0.08-0.17)
Seafood	0.00 (0-0.01)	0.02 (0-0.04)
Egg	0.00 (0-0)	0.01 (0-0.02)
Cheese	0.00 (0-0)	0.00 (0-0)
Plant-based soups	0.07 (0.05-0.1)	0.16 (0.12-0.2)
Vegetable and legume	0.06 (0.03-0.09)	0.11 (0.08-0.15)
Grain	0.01 (0.01-0.02)	0.05 (0.03-0.07)
Nuts and seeds	0.01 (0-0.01)	0.07 (0.04-0.1)
Grain-based dishes	0.78 (0.7-0.86)	3.14 (2.9-3.39)
Refined grain dishes	0.65 (0.56-0.74)	2.42 (2.19-2.65)
Breads[9]	0.06 (0.05-0.07)	0.60 (0.55-0.65)
Breakfast cereal	0.01 (0.01-0.01)	0.07 (0.05-0.08)
Bars[10]	0.01 (0-0.01)	0.05 (0.02-0.08)

Salty snacks[11]	0.01 (0.01-0.01)	0.16 (0.13-0.18)
Pancakes, waffles, and French toast[12]	0.01 (0-0.01)	0.04 (0.02-0.06)
Pasta	0.19 (0.13-0.25)	0.38 (0.25-0.51)
Rice	0.07 (0.06-0.08)	0.55 (0.42-0.68)
Mexican dishes[13]	0.17 (0.12-0.22)	0.23 (0.18-0.27)
Pizza[14]	0.13 (0.1-0.16)	0.35 (0.27-0.42)
Whole grain dishes	0.13 (0.06-0.21)	0.72 (0.59-0.85)
Breads[9]	0.01 (0.01-0.01)	0.10 (0.08-0.12)
Breakfast cereal	0.01 (0.01-0.01)	0.12 (0.1-0.14)
Bars[10]	0.00 (0-0.01)	0.05 (0.03-0.08)
Salty snacks[11]	0.02 (0.02-0.03)	0.33 (0.22-0.45)
Pancakes, waffles, and French toast[12]	0.00 (0-0)	0.00 (0-0)
Pasta	0.06 (-0.01-0.13)	0.03 (-0.01-0.07)
Rice	0.02 (0.01-0.02)	0.08 (0.06-0.1)
Mexican dishes[13]	0.00 (0-0.01)	0.00 (0-0)
Pizza[14]	0.00 (0-0)	0.00 (0-0)
Fruit	0.33 (0.29-0.36)	1.72 (1.53-1.92)
Whole	0.20 (0.17-0.23)	1.43 (1.23-1.63)
No added sugar or fruit juice[15]	0.19 (0.16-0.22)	1.37 (1.18-1.57)
With added sugar or fruit juice[15]	0.01 (0.01-0.01)	0.06 (0.04-0.07)
Dried	0.00 (0-0.01)	0.04 (0.02-0.06)
Juice	0.12 (0.11-0.14)	0.25 (0.22-0.29)
Juice, 100%[16]	0.11 (0.09-0.13)	0.23 (0.2-0.27)
Juice, with added sugar[16]	0.01 (0.01-0.02)	0.02 (0.01-0.03)
Vegetables	0.23 (0.2-0.26)	1.69 (1.47-1.92)
Dark green	0.02 (0.01-0.02)	0.16 (0.12-0.19)
Red and orange	0.02 (0.02-0.03)	0.20 (0.17-0.24)
Tomato juice	0.00 (0-0)	0.00 (0-0)
Potatoes	0.03 (0.02-0.04)	0.20 (0.16-0.24)
Other	0.16 (0.13-0.19)	1.13 (0.92-1.34)
Fats and oils	0.02 (0.02-0.03)	0.05 (0.04-0.06)
Table fats and spreads[17]	0.02 (0.01-0.02)	0.02 (0.01-0.02)
Vegetable oils	0.00 (0-0)	0.00 (0-0)
Salad dressings	0.01 (0.01-0.01)	0.03 (0.02-0.03)
Desserts	0.18 (0.15-0.22)	0.77 (0.63-0.92)
Cake and cookies[18]	0.08 (0.05-0.11)	0.48 (0.36-0.61)
Gelatin	0.01 (0-0.01)	0.02 (0.01-0.03)
Ice cream[19]	0.07 (0.05-0.09)	0.13 (0.09-0.17)
Non-dairy frozen[20]	0.00 (0-0)	0.01 (0-0.02)
Fruit-based	0.00 (0-0)	0.01 (0-0.01)
Candy	0.02 (0.02-0.03)	0.13 (0.09-0.16)
Beverages	0.88 (0.78-0.97)	2.08 (1.82-2.33)
Coffee and tea	0.09 (0.08-0.11)	0.50 (0.36-0.64)

Soft drinks	0.36 (0.31-0.42)	0.28 (0.24-0.32)
Soft drinks, with added sugar[21]	0.36 (0.31-0.42)	0.24 (0.2-0.28)
Soft drinks, no added sugar	0.00 (0-0)	0.04 (0.02-0.05)
Fruit-flavored	0.07 (0.05-0.09)	0.14 (0.04-0.23)
Energy	0.03 (0.01-0.04)	0.09 (0.04-0.15)
Nutrition drinks and meal replacements	0.08 (0.05-0.1)	0.08 (0.06-0.1)
Water, non-carbonated	0.02 (0.01-0.04)	0.18 (0.16-0.2)
Alcohol	0.23 (0.16-0.3)	0.80 (0.6-1)
Other[22]	0.11 (0.07-0.16)	0.30 (0.24-0.35)

All results were adjusted for kcal and survey cycle using linear regression models.

CO_2eq, carbon dioxide equivalent

[1]Predominant ingredient in mixed dish.

[2]Plant-based milk alternatives including soy milk, rice milk, and coconut milk.

[3]Excludes sandwich meats.

[4]Includes bacon, spareribs, cracklings, skin, and miscellaneous parts.

[5]Includes game meat.

[6]Plant-based meat alternatives made from legumes and grains.

[7]Includes egg-free frozen mix, dry mix, and liquid mix.

[8]Includes deli meats and and meat spreads.

[9]Includes yeast breads and quick breads such as biscuits, muffins, cornbread, popovers, and tortillas.

[10]Includes granola bars, cereal bars, and nutrition bars.

[11]Includes crackers, chips, popcorn, and pretzels.

[12]Includes crepes and funnel cakes.

[13]Includes burritos, tacos, quesadillas, enchiladas, chimichangas, chalupas, gorditas, fajitas, tamales, and

[14]Includes dumplings, turnovers, fritters, and knishes.

[15]Includes canned and frozen fruit.

[16]Includes fruit and vegetable juice blends.

[17]Includes butter, margarine, and sour cream.

[18]Includes pies and pastries.

[19]Includes gelato, sherbet, pudding, and custard.

[20]Includes ices and popsicles.

[21]Includes carbonated water with added sugar.

[22]Includes condiments, icing, sauces, gravies, jams/jellies, sweeteners, non-specified meats,

Supplemental Table 7: Mean daily greenhouse gas emissions and diet cost for the time restricted diet pattern, by food category, 2011-2018 (n=365)

Food and beverage category[1]	Greenhouse gas emissions (kg CO_2eq)	Cost (US $)
	Mean per day (95% CI)	
All foods	6.12 (5.55-6.68)	15.98 (14.62-17.34)
Dairy	0.44 (0.29-0.59)	0.99 (0.39-1.6)
Milk, fluid	0.29 (0.15-0.44)	0.62 (0.04-1.21)
Milk, dry	0.00 (0-0)	0.00 (0-0)
Cream	0.00 (0-0)	0.00 (0-0.01)
Cheese	0.11 (0.08-0.14)	0.24 (0.16-0.32)
Yogurt	0.03 (0-0.06)	0.11 (-0.02-0.24)
Milk and cream alternatives[2]	0.01 (0-0.01)	0.02 (0.01-0.04)
Protein dishes	1.69 (1.29-2.09)	3.91 (3.07-4.75)
Red meat	1.14 (0.76-1.53)	1.64 (0.87-2.41)
Beef[3]	1.07 (0.68-1.45)	1.03 (0.4-1.66)
Pork[3,4]	0.08 (0.05-0.11)	0.61 (0.29-0.93)
Lamb, goat, and veal[3,5]	0.00 (0-0)	0.00 (0-0)
Organ meat	0.00 (0-0)	0.00 (0-0)
Poultry[3]	0.20 (0.15-0.25)	1.23 (0.87-1.59)
Seafood[3]	0.23 (0.15-0.31)	0.88 (0.47-1.29)
Eggs	0.08 (0.05-0.11)	0.13 (0.07-0.2)
Plant proteins	0.04 (-0.03-0.1)	0.03 (0.01-0.05)
Meat alternatives[6]	0.00 (0-0)	0.00 (0-0)
Egg alternatives[7]	0.00 (0-0)	0.00 (0-0)
Beans, peas, and lentils	0.04 (-0.03-0.1)	0.03 (0.01-0.05)
Sandwiches and hotdogs	1.13 (0.8-1.47)	2.64 (1.78-3.5)
Hotdogs, sausages, and meat sandwiches[8]	1.13 (0.79-1.46)	2.54 (1.68-3.4)
Seafood sandwiches	0.01 (0-0.01)	0.09 (0.01-0.18)
Egg sandwiches	0.00 (0-0)	0.00 (0-0)
Nut butter sandwiches	0.00 (0-0)	0.00 (0-0.01)
Soups	0.04 (0-0.07)	0.13 (0.03-0.24)
Meat	0.01 (-0.01-0.03)	0.01 (-0.01-0.03)
Seafood	0.02 (-0.01-0.05)	0.08 (-0.02-0.17)
Egg	0.00 (0-0)	0.00 (0-0)
Cheese	0.00 (0-0)	0.00 (0-0)
Plant-based soups	0.01 (0-0.02)	0.05 (0.01-0.09)
Vegetable and legume	0.00 (0-0.01)	0.02 (0-0.04)
Grain	0.00 (0-0.01)	0.03 (-0.01-0.06)
Nuts and seeds	0.01 (0-0.01)	0.04 (0-0.09)
Grain-based dishes	0.96 (0.6-1.31)	2.78 (2.27-3.29)
Refined grain dishes	0.90 (0.54-1.26)	2.28 (1.78-2.77)
Breads[9]	0.05 (0.04-0.06)	0.53 (0.39-0.68)
Breakfast cereal	0.00 (0-0)	0.02 (0.01-0.04)

Bars[10]	0.00 (0-0)	0.00 (0-0)
Salty snacks[11]	0.01 (0.01-0.02)	0.20 (0.13-0.26)
Pancakes, waffles, and French toast[12]	0.01 (0-0.02)	0.03 (0-0.05)
Pasta	0.13 (0-0.26)	0.24 (0.13-0.36)
Rice	0.05 (0.02-0.07)	0.31 (0.05-0.57)
Mexican dishes[13]	0.46 (0.12-0.8)	0.45 (0.24-0.66)
Pizza[14]	0.19 (0.12-0.26)	0.49 (0.32-0.66)
Whole grain dishes	0.05 (0.03-0.08)	0.50 (0.29-0.72)
Breads[9]	0.00 (0-0.01)	0.06 (0-0.12)
Breakfast cereal	0.00 (0-0.01)	0.05 (0.02-0.08)
Bars[10]	0.00 (0-0)	0.03 (0-0.07)
Salty snacks[11]	0.03 (0.01-0.05)	0.31 (0.13-0.49)
Pancakes, waffles, and French toast[12]	0.01 (-0.01-0.02)	0.03 (-0.03-0.09)
Pasta	0.00 (0-0)	0.00 (0-0)
Rice	0.01 (0-0.01)	0.03 (0-0.05)
Mexican dishes[13]	0.00 (0-0)	0.00 (0-0)
Pizza[14]	0.00 (0-0)	0.00 (0-0)
Fruit	0.15 (0.1-0.2)	0.61 (0.43-0.79)
Whole	0.08 (0.05-0.11)	0.47 (0.3-0.64)
No added sugar or fruit juice[15]	0.07 (0.04-0.1)	0.45 (0.28-0.62)
With added sugar or fruit juice[15]	0.01 (0-0.01)	0.02 (0-0.03)
Dried	0.00 (0-0)	0.01 (0-0.02)
Juice	0.07 (0.03-0.11)	0.13 (0.07-0.2)
Juice, 100%[16]	0.06 (0.02-0.1)	0.12 (0.05-0.18)
Juice, with added sugar[16]	0.01 (0-0.02)	0.01 (0-0.03)
Vegetables	0.26 (0.15-0.37)	1.42 (0.99-1.85)
Dark green	0.00 (0-0.01)	0.03 (0-0.06)
Red and orange	0.02 (-0.01-0.04)	0.15 (0-0.29)
Tomato juice	0.00 (0-0)	0.00 (0-0)
Potatoes	0.09 (0.03-0.15)	0.42 (0.3-0.53)
Other	0.15 (0.05-0.24)	0.82 (0.43-1.21)
Fats and oils	0.03 (0.02-0.04)	0.08 (0.06-0.1)
Table fats and spreads[17]	0.01 (0.01-0.02)	0.03 (0.01-0.04)
Vegetable oils	0.00 (0-0)	0.00 (0-0)
Salad dressings	0.02 (0.01-0.02)	0.05 (0.04-0.07)
Desserts	0.20 (0.14-0.26)	0.92 (0.56-1.27)
Cake and cookies[18]	0.11 (0.06-0.16)	0.70 (0.36-1.03)
Gelatin	0.00 (0-0)	0.00 (0-0.01)
Ice cream[19]	0.07 (0.04-0.09)	0.09 (0.06-0.13)
Non-dairy frozen[20]	0.00 (0-0)	0.00 (0-0.01)
Fruit-based	0.00 (0-0)	0.00 (0-0.01)
Candy	0.02 (0.01-0.03)	0.12 (0.08-0.16)
Beverages	0.66 (0.52-0.8)	1.02 (0.87-1.18)

Coffee and tea	0.07 (0.01-0.13)	0.19 (0.12-0.26)
Soft drinks	0.46 (0.35-0.57)	0.35 (0.27-0.42)
Soft drinks, with added sugar[21]	0.46 (0.35-0.57)	0.33 (0.26-0.41)
Soft drinks, no added sugar	0.00 (0-0)	0.01 (0-0.02)
Fruit-flavored	0.06 (0.03-0.09)	0.14 (0.03-0.25)
Energy	0.02 (0-0.03)	0.04 (0-0.08)
Nutrition drinks and meal replacements	0.03 (0.01-0.05)	0.05 (0.02-0.07)
Water, non-carbonated	0.01 (0-0.01)	0.17 (0.13-0.2)
Alcohol	0.01 (0-0.02)	0.08 (0.02-0.15)
Other[22]	0.15 (0.04-0.26)	0.22 (0.14-0.3)

All results were adjusted for kcal and survey cycle using linear regression models.

CO_2eq, carbon dioxide equivalent

[1]Predominant ingredient in mixed dish.

[2]Plant-based milk alternatives including soy milk, rice milk, and coconut milk.

[3]Excludes sandwich meats.

[4]Includes bacon, spareribs, cracklings, skin, and miscellaneous parts.

[5]Includes game meat.

[6]Plant-based meat alternatives made from legumes and grains.

[7]Includes egg-free frozen mix, dry mix, and liquid mix.

[8]Includes deli meats and and meat spreads.

[9]Includes yeast breads and quick breads such as biscuits, muffins, cornbread, popovers, and tortillas.

[10]Includes granola bars, cereal bars, and nutrition bars.

[11]Includes crackers, chips, popcorn, and pretzels.

[12]Includes crepes and funnel cakes.

[13]Includes burritos, tacos, quesadillas, enchiladas, chimichangas, chalupas, gorditas, fajitas, tamales, and

[14]Includes dumplings, turnovers, fritters, and knishes.

[15]Includes canned and frozen fruit.

[16]Includes fruit and vegetable juice blends.

[17]Includes butter, margarine, and sour cream.

[18]Includes pies and pastries.

[19]Includes gelato, sherbet, pudding, and custard.

[20]Includes ices and popsicles.

[21]Includes carbonated water with added sugar.

[22]Includes condiments, icing, sauces, gravies, jams/jellies, sweeteners, non-specified meats,

Supplemental Table 8: Sensitivity analysis of mean daily greenhouse gas emissions with and without adjustment for losses and waste, 2011-2018 (n=8,146)

	Greenhouse gas emissions per day (kg CO_2eq)		
Diet pattern	TFD[1]	Consumed[1]	Diff. (%)
Plant-based	4.3 (4.0, 4.6)	2.7 (2.5, 2.9)	37
Low grain	6.5 (6.2, 6.9) [b]	4.2 (4.0, 4.4) [b]	36
Restricted carbohydrate	6.7 (6.5, 6.9) [b]	4.3 (4.2, 4.5) [b]	35
Low fat	5.8 (5.6, 6.0) [a]	3.7 (3.6, 3.9) [a]	36
Time restricted	6.1 (5.6, 6.7) [ab]	3.9 (3.5, 4.3) [ab]	36

[1]Mean (95% CI)

Diet patterns sharing a letter are not statistically different at P<0.005 using Wald tests (Bonferroni correction: 0.05 × 10 pairwise tests = 0.005).

All results were adjusted for kcal and survey cycle using linear regression models.

Total Food Demand (TFD) represents the sum of retail loss, consumer waste, inedible portions, and consumed food.

Diff. (%) represents the percent difference beetween Total Food Demand (TFD) and consumed food.

CO_2eq, carbon dioxide equivalent

Supplemental Table 9: Sensitivity analysis of mean daily diet costs with and without adjustment for losses, waste, and food away from home prices, 2011-2018 (n=8,146)

	FAH and FAFH prices							
Diet pattern	Purchased[1]		Consumed only[1]		Diff. (%)[2]	Consumed only (FAH prices only)[1]		Diff. (%)[3]
Plant-based	13.79 (13.10, 14.47)	a	8.66 (8.29, 9.04)		37	7.04 (6.75, 7.33)	a	19
Low grain	18.45 (17.04, 19.85)	bcd	12.50 (11.55, 13.44)	ab	32	7.96 (7.47, 8.44)	b	36
Restricted carbohydrate	20.79 (20.06, 21.52)	d	14.38 (13.85, 14.90)		31	9.30 (8.52, 10.08)	c	35
Low fat	18.74 (17.90, 19.58)	c	12.64 (12.08, 13.21)	b	33	8.64 (8.28, 9.01)	bc	32
Time restricted	15.98 (14.62, 17.34)	ab	10.90 (10.02, 11.79)	a	32	6.90 (6.61, 7.19)	a	37

[1]Mean (95% CI), values are US $.

[2]Percent difference between purchased and consumed food, including prices for foods at home and foods away from home.

[3]Percent difference between consumed food including prices for foods at home and foods away from home, and consumed food including food at home prices only.

Diet patterns sharing a letter are not statistically different at P<0.005 using Wald tests (Bonferroni correction: 0.05 × 10 pairwise tests = 0.005).

All results were adjusted for kcal and survey cycle using linear regression models.

Purchased food represents the sum of consumed food, consumer waste, and inedible portions.

CO_2eq, carbon dioxide equivalent

FAH, food at home

FAFH, food away from home

Supplemental Table 10: Sensitivity analysis of mean daily Healthy Eating Index-2015 scores with adjustment for demographic variables and non-mutually exclusive diet patterns, 2011-2018

Diet pattern	Model 1 (n=8,146)[1]		Model 2 (n=8,146)[2]		Model 3 (n=14,644)[3]	
	Mean per day (95% CI), HEI-2015 score					
Plant-based	48.0 (46.1, 49.9)	a	47.8 (46.0, 49.6)	ab	48.0 (46.0, 49.9)	a
Low grain	48.4 (47.1, 49.8)	a	49.7 (48.2, 51.2)	b	48.5 (47.1, 49.8)	a
Restricted carbohydrate	47.7 (46.9, 48.5)	a	47.3 (46.5, 48.0)	ab	48.5 (47.7, 49.3)	a
Low fat	52.8 (51.9, 53.7)		53.1 (52.2, 54.1)		52.5 (51.8, 53.1)	
Time restricted	43.7 (41.8, 45.7)		46.1 (43.9, 48.2)	a	45.1 (44.3, 46.0)	

Diet patterns sharing a letter are not statistically different at P<0.005 using Wald tests (Bonferroni correction: 0.05 × 10 pairwise tests = 0.005).

HEI-2015, Healthy Eating Index-2015

[1]Excludes participants that were categorized into >1 diet pattern. Adjusted for kcal and survey cycle using linear regression models.

[2]Excludes participants that were categorized into >1 diet pattern. Adjusted for kcal, survey cycle, age, sex, education, income-to-poverty ratio, and race/ethnicity using linear regression models.

[3]Includes participants that were categorized into >1 diet pattern. Adjusted for kcal and survey cycle using linear regression models.

Supplemental Table 11: Sensitivity analysis of mean daily greenhouse gas emissions with adjustment for demographic variables and non-mutually exclusive diet patterns, 2011-2018

Diet pattern	Model 1 (n=8,146)[1]		Model 2 (n=8,146)[2]		Model 3 (n=14,644)[3]	
	Mean per day (95% CI), kg CO_2eq					
Plant-based	4.3 (4.0, 4.6)		4.4 (4.1, 4.6)		3.9 (3.7, 4.2)	
Low grain	6.5 (6.2, 6.9)	b	6.5 (6.2, 6.8)	b	5.8 (5.5, 6.1)	ab
Restricted carbohydrate	6.7 (6.5, 6.9)	b	6.7 (6.5, 6.9)	b	6.3 (6.1, 6.5)	c
Low fat	5.8 (5.6, 6.0)	a	5.8 (5.6, 6.1)	a	5.5 (5.4, 5.7)	ab
Time restricted	6.1 (5.6, 6.7)	ab	6.0 (5.4, 6.6)	ab	6.0 (5.8, 6.2)	bc

Diet patterns sharing a letter are not statistically different at P<0.005 using Wald tests (Bonferroni correction: 0.05 × 10 pairwise tests = 0.005).

[1]Excludes participants that were categorized into >1 diet pattern. Adjusted for kcal and survey cycle using linear regression models.

[2]Excludes participants that were categorized into >1 diet pattern. Adjusted for kcal, survey cycle, age, sex, education, income-to-poverty ratio, and race/ethnicity using linear regression models.

[3]Includes participants that were categorized into >1 diet pattern. Adjusted for kcal and survey cycle using linear regression models.

Supplemental Table 12: Sensitivity analysis of mean daily diet cost with adjustment for demographic variables and non-mutually exclusive diet patterns, 2011-2018

Diet pattern	Model 1 (n=8,146)[1]		Model 2 (n=8,146)[2]		Model 3 (n=14,644)[3]	
	Mean per day (95% CI), US $					
Plant-based	13.8 (13.1, 14.5)	a	13.8 (13.1, 14.5)		12.7 (12.0, 13.4)	
Low grain	18.4 (17.0, 19.9)	bcd	19.1 (17.6, 20.7)	ab	16.4 (15.3, 17.5)	a
Restricted carbohydrate	20.8 (20.1, 21.5)	d	20.6 (19.9, 21.4)	b	19.8 (19.2, 20.3)	
Low fat	18.7 (17.9, 19.6)	c	19.1 (18.2, 20.0)	ab	17.4 (16.8, 18.0)	a
Time restricted	16.0 (14.6, 17.3)	ab	17.0 (15.6, 18.4)	a	17.0 (16.0, 17.9)	a

Diet patterns sharing a letter are not statistically different at P<0.005 using Wald tests (Bonferroni correction: 0.05 × 10 pairwise tests = 0.005).

[1]Excludes participants that were categorized into >1 diet pattern. Adjusted for kcal and survey cycle using linear regression models.

[2]Excludes participants that were categorized into >1 diet pattern. Adjusted for kcal, survey cycle, age, sex, education, income-to-poverty ratio, and race/ethnicity using linear regression models.

[3]Includes participants that were categorized into >1 diet pattern. Adjusted for kcal and survey cycle using linear regression models.
